# Extracellular vesicles as nanotheranostic platforms for targeted neurological disorder interventions

**DOI:** 10.1186/s40580-024-00426-5

**Published:** 2024-05-13

**Authors:** Hye Kyu Choi, Meizi Chen, Li Ling Goldston, Ki-Bum Lee

**Affiliations:** Department of Chemistry and Chemical Biology, The State University of New Jersey, 123 Bevier Road, Rutgers, Piscataway, NJ 08854 USA

**Keywords:** Central nervous system (CNS) diseases, Nanotechnology, Extracellular vesicles (EVs), CNS theranostics, Diagnostic nanotools, Early diagnosis of neurological disorders, EV-based therapeutics

## Abstract

Central Nervous System (CNS) disorders represent a profound public health challenge that affects millions of people around the world. Diseases such as Alzheimer’s disease (AD), Parkinson’s disease (PD), and traumatic brain injury (TBI) exemplify the complexities and diversities that complicate their early detection and the development of effective treatments. Amid these challenges, the emergence of nanotechnology and extracellular vesicles (EVs) signals a new dawn for treating and diagnosing CNS ailments. EVs are cellularly derived lipid bilayer nanosized particles that are pivotal in intercellular communication within the CNS and have the potential to revolutionize targeted therapeutic delivery and the identification of novel biomarkers. Integrating EVs with nanotechnology amplifies their diagnostic and therapeutic capabilities, opening new avenues for managing CNS diseases. This review focuses on examining the fascinating interplay between EVs and nanotechnology in CNS theranostics. Through highlighting the remarkable advancements and unique methodologies, we aim to offer valuable perspectives on how these approaches can bring about a revolutionary change in disease management. The objective is to harness the distinctive attributes of EVs and nanotechnology to forge personalized, efficient interventions for CNS disorders, thereby providing a beacon of hope for affected individuals. In short, the confluence of EVs and nanotechnology heralds a promising frontier for targeted and impactful treatments against CNS diseases, which continue to pose significant public health challenges. By focusing on personalized and powerful diagnostic and therapeutic methods, we might improve the quality of patients.

## Introduction

**Fig. 1:**
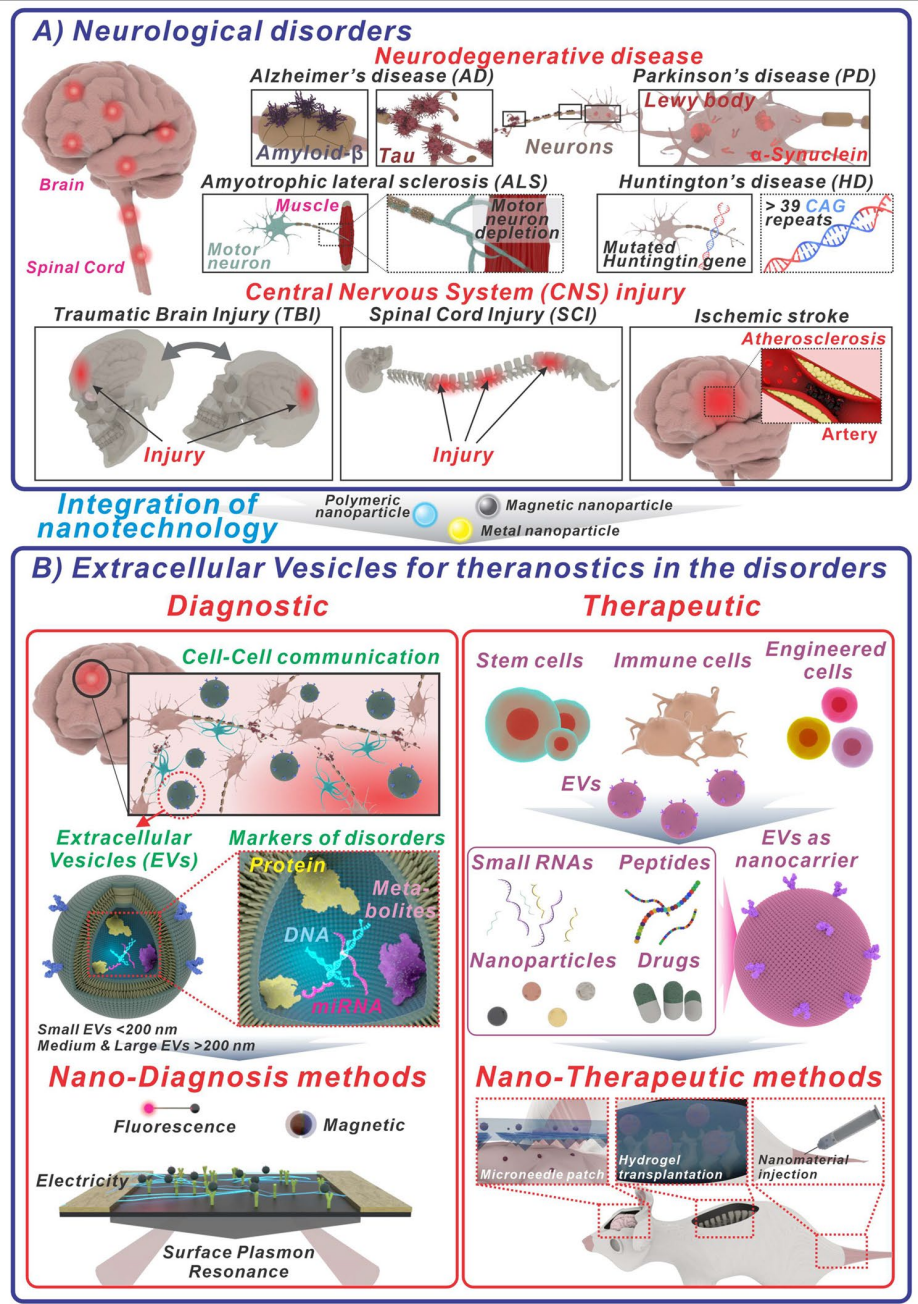
Schematic diagram of neurological disorders and how EVs can be used in nanotechnology-assisted theranostics. **(A)** neurological disorders that can occur in the CNS, such as the brain and spinal cord are illustrated. Two types of representative disorders are shown: neurodegenerative diseases (AD, PD, ALS, and HD) and CNS injuries (TBI, SCI, and ischemic stroke). **(B)** The diagram shows how EVs can be used for the theranostics of neurological disorders. The EVs in CNS cells naturally carry biomarkers such as proteins, DNA, miRNA, and metabolites, which are vital for intercellular communication. Nano detection of EVs, therefore, can be a promising approach for the diagnosis of neurological disorders. Additionally, EVs can also be used as nanocarriers for therapeutic agents. Native EVs extracted from cells containing their own signature biomolecules can be used for nano-therapeutic methods such as microneedle patches, hydrogel transplantation, and nanomaterial injection in vivo

Central nervous system (CNS) diseases pose a significant challenge to global public health, affecting both individuals and societies [1, 2]. The diseases encompass various severe conditions, including neurodegenerative disorders like Alzheimer’s disease (AD) [3, 4] and Parkinson’s disease (PD) [5, 6], as well as traumatic brain injuries (TBI) [7] and spinal cord injuries (SCI) [8]. Each condition presents unique symptoms affecting cognitive function, movement, sensation, and emotional health [9, 10]. The impact of these diseases extends beyond the individuals diagnosed, placing a considerable burden on healthcare systems striving to provide effective treatment and support [Fig. 1a]. Each disorder within the spectrum of CNS diseases presents its own set of unique symptoms and underlying causes, which can complicate the processes of diagnosis and treatment [11–14].

In response to these challenges, biomedical researchers are deeply engaged in investigating the fundamental pathways and scientific mechanisms that contribute to the onset of these diseases to enhance our comprehension and develop more effective therapeutic interventions. For instance, certain CNS disorders, such as AD and PD, have been linked to the pathological accumulation of specific proteins within the brain. This process disrupts normal brain function [15]. Conversely, Amyotrophic Lateral Sclerosis (ALS) is characterized by the progressive degeneration of motor neurons, leading to severe muscle weakness and loss of control [16]. TBI and SCI are usually the result of direct physical trauma to the brain and spinal cord, respectively, resulting in a wide range of functional impairments [10]. In their quest to unravel the complexities of CNS diseases, researchers have achieved notable advancements in understanding their etiology and formulating potential treatments [17, 18]. One significant stride has been the identification of biomarkers that can facilitate the early detection and diagnosis of these conditions [15]. Advanced brain imaging technologies, such as Magnetic Resonance Imaging (MRI) and Positron Emission Tomography (PET) scans, have proven instrumental in revealing structural alterations in the brain that could signify the development of a CNS disorder [19]. The primary therapeutic strategies for managing CNS diseases typically involve interventions for the regions affected, including pharmacological treatments to address biochemical imbalances or surgical procedures to mitigate physical damage.

However, one of the biggest hurdles to managing CNS diseases is the difficulty of diagnosing them early. Many of these diseases exhibit subtle symptoms or are asymptomatic in their initial stages, making it difficult for healthcare professionals to diagnose them promptly [15, 20]. As a result, treatment often begins only after the disease has progressed significantly, limiting the effectiveness of interventions. CNS diseases often present with a wide range of symptoms that vary among individuals. This heterogeneity makes it challenging to develop standardized diagnostic criteria and treatment approaches [21, 22]. What works for one patient may not be effective for another, necessitating personalized treatment strategies tailored to each patient’s unique symptoms and disease progression. Although some treatments are available to manage symptoms and slow disease progression, there remains a significant unmet need for curative therapies for many CNS diseases. The complexity of the CNS, coupled with the blood-brain barrier (BBB), poses challenges to deliver therapeutics to affected regions of the brain or spinal cord effectively [23, 24].

To this end, the convergence of nanotechnology and theranostics may offer a paradigm shift in disease management, exhibiting immense potential for revolutionizing traditional therapeutic and diagnostic approaches.

Nanotechnology, which focuses on manipulating materials at the nanoscale through innovative fabrication techniques, offers immense potential for advancements in theranostics [25–28]. This technology can further harness the unique properties of materials at the nanoscale, particularly in the electrical, optical, and chemical domains, to create novel tools for both diagnosis and treatment of disease [29–31]. They also have substantial surface area-to-volume ratios, which makes them ideal for drug modification or attachment of detection probes [32–34]. By employing nano-sized patterned platforms, researchers have successfully detected markers that serve as indicators for CNS diseases. This platform has shown remarkable efficacy in early disease marker detection, marking a significant step toward early intervention and treatment [35, 36]. However, the nanotechnology-based theranostic platform still faces several challenges that need to be resolved. Refining targeting mechanisms is essential to ensure the precise delivery of therapeutic agents to affected sites, optimizing treatment efficacy while mitigating off-target effects. Improving the sensitivity and specificity of methods for early disease detection is essential, as it facilitates timely medical intervention and has the potential to significantly modify the course of the disease. Moreover, addressing concerns regarding invasiveness in diagnostic and therapeutic approaches is significant. Minimizing the invasiveness of procedures is critical to reducing patient discomfort and complications while improving overall treatment outcomes.

One prominent example of nanotechnology’s potential in theranostics lies in exploring extracellular vesicles (EVs). These naturally occurring, nanoscale membrane-bound particles hold immense promise for revolutionizing the diagnosis and treatment of CNS disease. These are membranous vesicles that are secreted by all cell types, such as healthy, diseased, stem, and transformed cells [37, 38]. There are three major categories of EVs, namely exosomes, microvesicles, and apoptotic bodies, which are classified based on their biogenesis mechanism [39, 40]. The CNS uses both synaptic communication and EVs for intercellular communication [41, 42]. Unlike synaptic vesicles, EVs can facilitate communication over longer distances and transfer information between various cells within the CNS [43, 44]. EVs are proficient at carrying diverse biomolecules, such as proteins, messenger RNAs (mRNAs), and non-coding RNAs, to target cells [45–47]. This results in various functional impacts on these recipient cells. EVs play critical roles in maintaining neuronal homeostasis. However, EVs from dysfunctional nerve cells can have negative effects, possibly contributing to the pathophysiology of different neurological conditions [45, 48]. Hence, it’s crucial to strategically utilize the beneficial attributes of EVs while neutralizing their harmful effects when developing treatments for neurological disorders. A more profound understanding of the specific biomolecular cargo and the mechanisms of EV-mediated cellular communication within the CNS could reveal novel nanotherapeutic approaches for treating neurological diseases. They deliver a payload of proteins, mRNAs, and non-coding RNAs to acceptor cells [45], inducing functional effects on the recipient cells. EVs are essential in maintaining neuronal homeostasis, but EVs from dysfunctional CNS cells may exert detrimental effects [45, 48]. Harnessing the beneficial functions of EVs while mitigating their detrimental effects is crucial for developing interventions for neurological disorders. The exploration and comprehension of the specific cargo and mechanisms involved in EV-mediated communication within the CNS hold great potential for unveiling new nanotherapeutic strategies to combat neurological diseases.

From this point of view, the integration of EVs with nanotechnology has enormous potential to overcome the limitations encountered when utilizing either approach alone in theranostics for CNS diseases [49–51]. On the one hand, nanotechnology provides a sophisticated toolkit for manipulating and engineering EVs to enhance their therapeutic and diagnostic capabilities [52]. Nanoparticles can capture EVs, improving their stability and bioavailability. Surface modification techniques enable precise targeting of EVs to specific cell types or regions within the CNS, enhancing therapeutic efficacy while minimizing off-target effects. Additionally, nanotechnology-based imaging modalities allow for real-time monitoring of EV distribution and uptake, facilitating accurate assessment of treatment response and disease progression. On the other hand, EVs offer several advantages over traditional nanocarriers. Their natural origin and biocompatibility reduce the risk of immune reactions and toxicity [53]. In addition, EVs have inherent targeting capabilities and can cross the BBB, facilitating efficient delivery of therapeutic cargo to the CNS [54–57]. By harnessing EVs as carriers, nanotechnology-based theranostic platforms can leverage these unique properties to overcome the challenges associated with drug delivery to the brain. Furthermore, the cargo carried by EVs, including proteins, mRNAs, and non-coding RNAs, can be precisely engineered or modified using nanotechnology techniques [58, 59]. This allows for tailored delivery of therapeutic payloads to diseased cells, maximizing therapeutic efficacy while minimizing side effects [Fig. 1b].

Overall, integrating EVs with nanotechnology offers a synergistic approach to theranostics for CNS diseases, combining the advantages of both platforms to overcome the limitations encountered when using either approach alone. By leveraging the unique properties of EVs and the sophisticated capabilities of nanotechnology, researchers can develop innovative strategies for the diagnosis, treatment, and management of neurological disorders. In this review, we explore nanotechnology-assisted EVs for the theragnostic study of CNS diseases. In our overview, we cover a range of prevalent CNS diseases, such as AD, PD, ALS, Huntington’s disease (HD), TBI, and SCI. We highlight the urgent need for novel diagnostic and therapeutic approaches by outlining the diverse spectrum of these neurological disorders. Next, we explain the critical roles of EVs in detecting and treating CNS diseases. Within the CNS, various types of EVs, such as exosomes, microvesicles, and apoptotic bodies, play a crucial role in facilitating intercellular communication. Their ability to transport multiple molecular cargo, including proteins, mRNAs, and non-coding RNAs, makes them valuable for coordinating cellular responses and maintaining neuronal homeostasis. Additionally, we explore how EVs can cross the BBB and facilitate communication over long distances, making them essential vehicles for targeted drug delivery and biomarker detection in the CNS microenvironment. Finally, we present a comprehensive survey of recent research endeavors that combine EVs and nanotechnology for CNS theranostics. In various in vitro and in vivo experiments, we describe how researchers have cleverly leveraged nanotechnology to improve the diagnostic sensitivity and therapeutic efficacy of EV-based therapies. Through the encapsulation of therapeutic agents within nanocarriers or by engineering the surfaces of EVs for targeted delivery, researchers have made significant strides in slowing disease progression and improving neurological outcomes. This review also focuses on interdisciplinary expertise and incorporates recent discoveries to provide new perspectives and promote cutting-edge nano-theranostic approaches for CNS diseases. By narrowing the divide between foundational research and its clinical application, we aim to lay the groundwork for breakthroughs in diagnosing and treating CNS disorders. Such advancements hold the promise of providing significant relief and new hope to those suffering from these challenging and often incapacitating conditions.

## Neurological disorders

CNS disorders are devastating maladies of the twenty-first century typically characterized by damage to neurons. Due to the inability of neurons to regenerate, patients suffering from CNS damage often experience neurological decline, physical disability, and even death. When considering the various disorders that can affect the CNS, it becomes evident that neurodegeneration and acute CNS injury pose substantial burdens in the healthcare system.

### Neurodegenerative diseases

Neurodegenerative diseases encompass various illnesses that are characterized by the progressive loss of neurons [60]. The broad classifications of neurodegenerative diseases include extrapyramidal and pyramidal movement disorders and cognitive or behavioral disorders. Furthermore, hallmarks of neurodegenerative diseases typically include protein abnormalities, with amyloidoses, tauopathies, a-synucleinopathies, and TDP-43 proteinopathies being the most common. Despite the pathological distinctions of neurodegenerative diseases, processes shared by all subsets include neuroinflammation, proteotoxic stress, and oxidative stress [60]. The characteristics previously discussed are shared across various forms of neurodegeneration, accompanying the dysfunction and eventual demise of neuron cells. As life expectancy increases and the elderly population expands, the incidence of neurodegenerative diseases is expected to surge in the forthcoming years. This trend is likely to lead to a significant increase in disability and mortality rates associated with these conditions.

#### AD

AD is the most common neurodegenerative disease, and the most common form of dementia, comprising 60–70% of all dementia cases [61]. AD is a slowly progressing neurodegenerative disease, with affected patients typically experiencing episodic memory impairment followed by cognitive decline such as language difficulties, decline in visuospatial function, and dementia [15]. There are two main histopathological features in AD brains: first, the accumulation of extracellular senile plaques by the deposition and aggregation of amyloid-β (Aβ), and second, the intracellular neurofibrillary tangles (NFTs) of hyperphosphorylated tau (p-Tau) [62]. Although the presence of senile plaques is not uncommon in a healthy aging brain, the Aβ present in AD pathology is typically formed from the dysfunction of the transmembrane amyloid precursor protein (APP) proteolytic cleavage. APP is cleaved by β-APP cleaving enzyme (BACE1) followed by β-and γ-secretases (presenilin-1 and presenilin-2, respectively), resulting in the formation of Aβ. The two main isoforms of Aβ are Aβ40 (40 amino acid residues long) and Aβ42 (42 amino acid residues long). Despite the higher concentration of Aβ40 found in the cerebral spinal fluid of AD patients, Aβ42 is the major component of senile plaques due to its greater propensity to aggregate [63]. Similar to Aβ presence in a healthy aging brain, tau-containing tangles are present in the brains of most healthy people over the age of 60 years old. However, these tangles are predominantly restricted to the medial temporal lobe. In AD pathology, these tau-containing tangles accumulate in considerable quantities elsewhere in the neocortex [15]. The main role of Tau, a protein associated with microtubules, is to stabilize microtubules, specifically in axons. In the case of AD, it is observed that any of the six tau isoforms can potentially undergo hyperphosphorylation within their proline-rich regions. This, in turn, leads to the promotion of aggregation and the formation of NFTs [15].

Most AD cases are late-onset sporadic cases with no known genetic association. However, studies have shown that autosomal dominant genes increase the risk of developing AD, making the heritability of AD approximately 70% [64, 65]. The most common genetic mutations associated with early-onset AD include mutations in β- APP, presenilin 1 (PSEN1), and presenilin 2 (PSEN2). It has also been determined that the ε4 allele of the apolipoprotein E (APOE) gene has also been determined to increase the risk of AD due to the high affinity of APOEε4 for Aβ, thus accelerating fibril formation [66]. Despite the increasing incidence of AD worldwide, AD remains challenging to diagnose early due to the late onset of clinical symptoms, such as memory loss and cognitive decline. Histopathological changes such as Aβ deposition and aggregation begin to occur in the brain over a decade before the manifestation of clinical symptoms [67, 68]. A definitive diagnosis of AD only occurs through post-mortem brain tissue evaluation. However, several methods have been employed to assess suspected AD in living patients [69, 70]. Besides presenting clinical symptoms, PET scans of Aβ plaques in the brain can be used to diagnose AD with high accuracy (96% sensitivity and 100% specificity) [71]. However, due to the high cost of this imaging method, amyloid-specific PET testing is not a widely implemented diagnostic tool. A cheaper, more common method for AD diagnosis is evaluating disease-related biomarkers in biofluids. Biomarkers include Aβ42, p-tau, and total tau protein content in cerebrospinal fluid (CSF). Although relatively cheaper than imaging techniques, CSF evaluation has drawbacks for patients, including the invasive nature of lumbar puncture and the long turnaround time for results [70]. Currently, available treatments for AD only treat symptoms, and there are only four approved drugs by the Food and Drug Administration (FDA) for AD [72]. Three of the drugs are acetylcholinesterase (AChE) inhibitors (donepezil, galantamine, and rivastigmine), and one drug is an N-methyl-D-aspartate (NMDA) receptor antagonist (memantine) [73, 74]. Studies have indicated that the effectiveness of treatment is significantly higher when a combination therapy is employed, incorporating both types of drugs, compared to treatments that do not utilize a combination approach [75].

#### PD

PD is the second most common neurodegenerative disease worldwide after AD, with approximately 6.1 million affected patients in 2016 [5]. PD is a slowly progressing neurodegenerative disease and movement disorder that typically affects the elderly population. The most common clinical symptoms of PD include motor symptoms, such as bradykinesia, muscular rigidity, resting tremor, postural instability, and gait impairment [76]. Although these symptoms are strongly associated with PD, non-motor symptoms typically occur 5–10 years before the onset of motor features [77]. Common non-motor symptoms include constipation, REM sleep behavior disorder, depression, shoulder pain, and hyposmia [5, 15]. Due to the non-specific nature of the aforementioned non-motor features, it is difficult for clinicians to recognize and suspect PD in the early stage. The pathological changes in the CNS responsible for the motor symptoms of PD patients is the loss of dopaminergic neurons in the substantia nigra pars compacta region of the midbrain. Neuronal death is caused by the aggregation of the misfolded alpha-synuclein (α-syn) protein inside of the neurons in the form of Lewy bodies and Lewy neurites. α-syn is a protein that affects the release of synaptic vesicles and neurotransmitters [5, 15].

Similar to AD, most cases of PD are sporadic with no genetic component. However, several genetic mutations have been linked to monogenic forms of PD and earlier onset PD. Most of the interest in monogenic PD research involves mutations in the SNCA, LRRK2, PRKN, PINK1, and GBA genes. The SNCA gene encodes α-syn, and all mutations in the SNCA gene are associated with an earlier age of PD onset. Mutations in LRRK2 (Gly2019Ser) result in a disease phenotype identical to sporadic PD. The PRKN and PINK1 genes are involved in mitochondrial and mitophagy function, and mutations in these genes are strongly associated with autosomal recessive and early-onset PD. Mutations in the GBA gene are associated with earlier age of PD onset and rapid cognitive decline [5, 78]. Despite the overwhelming majority of all PD cases having no genetic association, great interest exists in the research of gene-targeting treatments for monogenic PD.

The diagnosis of PD is based on a history, physical examination, and the exclusion of alternative causes. The International Parkinson and Movement Disorder Society proposed a revised set of criteria for PD diagnosis that enhances the diagnostic accuracy of the conventionally used Queen’s Square Brain Bank Criteria. For PD diagnosis, an individual must present Parkinsonian syndrome, defined as the presence of bradykinesia with resting tremor, rigidity, or both. The diagnostic criteria also require individuals to present supportive and exclusionary features [6, 79]. Advanced imaging techniques such as Dopamine transporter single-photon emission computed tomography (DaT SPECT) can be used for PD diagnosis by confirming the presynaptic dopamine neuronal dysfunction in PD. Treatment of PD is symptomatic, with pharmacologic treatments of PD motor symptoms being dopamine-based and pharmacologic treatments of PD non-motor symptoms being based on other neurotransmitters such as serotonin and acetylcholine [79]. As with AD, none of the available pharmacological treatments for PD can modify disease progression.

#### Other (ALS and HD)

Besides AD and PD, two other notable neurodegenerative diseases include ALS and HD. ALS is a neurodegenerative disease characterized by degeneration of upper and lower motor neurons, resulting in paralysis and death. Two main types of initial symptoms occur in ALS patients: spinal-onset disease and bulbar-onset disease. Patients with spinal-onset disease experience muscle weakness of the limbs, whereas patients with bulbar-onset disease typically experience dysarthria (difficulty with speech) and dysphagia (difficulty swallowing) [80]. In addition to the aforementioned symptoms, about 50% of ALS patients’ experience cognitive impairment. The prominent histopathological feature of ALS is the aggregation and accumulation of ubiquitinylated proteins, most commonly TAR DNA-binding protein 43 (TDP43) in motor neurons. At the cellular level, spinal motor neuron depletion occurs, along with the atrophy of skeletal muscles and motor cortex [80]. The diagnosis of ALS is based on the presence of clinical symptoms and motor neuron dysfunction, as well as the exclusion of an alternative diagnosis. Commonly, electrophysiology tests are performed to confirm malfunction in the lower motor neurons. There is no cure for ALS, and care involves a multidisciplinary team of specialists and healthcare professionals to improve the quality of life for the patient [81].

HD is the most common autosomal dominant neurodegenerative disorder. This disease is caused by an inherited CAG trinucleotide repeat expansion in the Huntingtin (HTT) gene, producing a mutant Huntingtin protein with a long polyglutamine repeat. If the HTT contains > 39 CAG repeats, HD is fully penetrant, and the patient is certain to develop HD. Typically, HD manifests in patients around 30–40 years of age and presents with various motor and cognitive disturbances [82, 83]. Diagnosis of HD depends on genetic tests and the presentation of motor symptoms, as outlined by the Unified HD Rating Scale (UHDRS) total motor score (TMS) diagnostic confidence score. As with ALS, HD care requires a multidisciplinary team to optimize patients’ quality of life [82].

### CNS injury

#### TBI

TBI, which represents a type of acute injury to the CNS, holds the distinction of being the most prevalent among all neurological disorders [84]. Annually, it is estimated that between 50 and 60 million individuals globally suffer from a TBI [13]. Projections indicate that TBI will continue to be among the top three causes of injury-related death and disability until at least 2030, underscoring the expectation that the extensive worldwide impact of TBI will increase even further [85]. TBI has long-lasting consequences beyond the initial incident, such as years of lost life (YLLs) and years lived with disability (YLDs). According to the Global Burden of Disease (GBD) report, in 2016, TBIs resulted in 8.1 million YLDs [13]. While YLLs due to TBIs have been reported for certain countries or populations, data from 16 European countries suggests that each TBI death is associated with about 24 YLLs. This translates to an age-standardized rate of roughly 160 YLLs per 100,000 [86]. In New Zealand, estimates indicate that there were 20,300 disability-adjusted life years (DALYs) attributed to TBI, representing 27% of total injury-related health loss and 2.4% of DALYs from all causes. Of these, 71% of TBI DALYs resulted from fatal TBIs [87]. TBI refers to any brain injury that results in damage to brain tissues and can be divided into three categories: closed head, penetrating, and explosive blast TBI. The incidence of closed-head TBI is the highest and is commonly caused by motor vehicle accidents, falls, and acts of violence. The clinical symptoms of TBI vary between patients, and common features include coma, headache, nausea, seizures, amnesia, and behavioral abnormalities [88, 89]. Damage to the brain that occurs during TBI includes (i) primary injury, direct damage resulting from mechanical injury, and (ii) secondary injury, which is indirect cellular damage following primary injury. During primary injury, the initial mechanical insult can cause focal and diffuse brain injuries. Focal brain injuries commonly occur in closed brain TBI and penetrating TBI, and features include skull fracture as well as localized contusion at the injury site. Unlike focal brain injury, diffuse injury results from the force of rapid acceleration/ deceleration that can cause shearing and stretching injuries to the tissues of the brain. The resultant damage to the neuronal axons and blood vasculature can lead to brain edema and ischemia [89].

After the primary injury occurs, the insult induces a series of indirect damages to the brain that comprise the secondary injury. Various cellular and molecular factors drive the secondary injury, including excitotoxicity, mitochondrial dysfunction, oxidative stress, neuroinflammation, glial scar, and cell apoptosis. Excitotoxicity is caused by the excessive release of excitatory amino acids such as glutamate due to initial neuronal cell death and disruption of the BBB. The increased presence of glutamate activates ion channels, causing an abnormal influx of ions such as Na^+^, K^+^, and Ca^2+^ into neurons, resulting in neuronal membrane depolarization [89]. Moreover, activation of ion channels such as the N-methyl-d-aspartate (NMDA) receptor has been shown to promote the production of reactive oxygen species (ROS) [90, 91]. Both alterations in ion homeostasis and accumulation of ROS play a role in causing mitochondrial dysfunction. As a result of the excessive ion influx into the mitochondria, the mitochondrial membrane is depolarized, and more ROS is produced. This causes dysregulation of cellular metabolism and ultimately, cell death, while the continued accumulation of ROS causes oxidative stress. Subsequent oxidative stress damages proteins and DNA and causes lipid peroxidation, thus compromising cellular membranes. Acute TBI may initially appear mild at admission but can progress to a more severe state with varying delays. The injury can occur due to multiple reasons and is often a result of secondary injuries [92]. Additionally, individuals who have experienced previous TBIs, such as those who have had repeated injuries while engaging in contact sports, are less likely to achieve full recovery from an index TBI [93]. During the acute secondary injury phase, 24 h post primary injury, the damaged BBB allows an influx of immune cells such as neutrophils, monocytes, and lymphocytes into the injured region. These infiltrating immune cells secrete pro-inflammatory cytokines, including tumor necrosis factor (TNF)-α, interleukin (IL)-1β, and IL-6, which drive neuroinflammation. Besides the activation of immune cells during TBI, astrocytes are activated, infiltrate the injury site, and undergo reactive astrogliosis. Activated astrocytes contribute to a scar-like structure known as the glial scar in coordination with cells, such as microglia and fibroblasts. Glial scar formation poses a significant obstacle to the regrowth of axons in TBI cases. The final major feature of secondary injury is the apoptotic cell death of neurons and oligodendrocytes. This apoptotic cell death results from various cellular and molecular pathways and the interaction of pro-inflammatory cytokines such as TNF-α with their cellular receptors [89]. With the multitude of deleterious effects occurring in the TBI brain, the need to diagnose and treat TBI promptly is crucial.

In acute medical care, non-contrast computed tomography (CT) imaging is most commonly used to diagnose TBI. CT can be performed quickly and is effective in diagnosing both extra- and intra-axial hemorrhage. MRI possesses superior diagnostic sensitivity for non-hemorrhagic injuries [94]. Treatments for TBI include head elevation and hyperventilation, both methods that reduce intracranial pressure for the injured patient. Current TBI treatment guidelines also allow seven days of prophylactic antiepileptics to help prevent early seizures. Additional medical interventions for treating TBI encompass strategies such as therapeutic hypothermia, also known as therapeutic cooling, or inducing a medically controlled unconscious state in the patient. These approaches aim to decrease the brain’s metabolic demands, thereby mitigating further damage. In more severe cases of TBI, where there is a significant mass effect resulting from a hematoma or contusion, surgical intervention becomes imperative. This can involve procedures such as a craniotomy, where a portion of the skull is removed to access the brain, or a decompressive craniectomy, which involves removing a section of the skull to alleviate pressure on the brain. These surgical measures are critical for relieving pressure within the skull and preventing additional brain injury [95].

#### SCI

SCI refers to damage to the spinal cord that causes changes in its function. Although SCI is not as common an acute CNS injury as TBI, an estimated 250,000–500,000 people globally suffer a SCI each year [8, 96, 97]. The impact of spinal cord injuries (SCI) on patients and their families is significant and multifaceted. This includes the physical, social, psychological, and financial burden that accompanies SCI. Given that SCI often results in lifelong physical dependence and disability, the challenges faced by patients and their families are profound and long-lasting. Accompanying the increased lifelong mortality rate, SCI patients can expect to pay $1.1–4.6 million USD on average in direct medical costs during their lifetime. The incidence of SCI has increased considerably in the last 30 years, with the global prevalence of SCI increasing from 236 to 1298 cases per million people [97]. The cause of the SCI distinguishes the two main types of SCI: non-traumatic and traumatic SCI. Non-traumatic SCI results from disease processes such as tumors, infections, or degenerative disc disease that causes damage to the spinal cord. On the contrary, traumatic SCI results from physical impact, usually caused by motor vehicle accidents, falls, sports injuries, and violence [96].

In the case of traumatic SCI, external mechanical impact results in primary and secondary injury. During the primary injury phase, the initial impact causes bone fractures and dislocation of the vertebrae, which can compress or transect the spinal cord. Cells at the focal injury site, including neurons and oligodendrocytes, are damaged in this phase and disrupted vasculature results in the formation of hemorrhage and the disruption of the blood-spinal cord barrier. The primary injury triggers the secondary injury, which rapidly ensues. The secondary injury phase can be subdivided into acute (< 48 h), subacute (48 h to 14 days), and intermediate-chronic (14 days to > 6 months) phases based on the time after primary injury. During the acute phase of secondary injury, cell permeabilization, apoptotic signaling, and cell death rapidly occur due to damaged vasculature. Furthermore, resulting hemorrhaging causes an increase in pro-inflammatory cytokines such as TNF-α and IL-1β within minutes of primary injury. In parallel, inflammatory cells such as macrophages, neutrophils, and leukocytes influx into the injury region. Due to the intense inflammatory response, the spinal cord continues to swell and compress, further worsening the injury [96]. During the subacute secondary injury phase, ischemia and excitotoxicity cause cell death, and free radical production occurs. Moreover, activated immune cells enhance the inflammatory response and contribute to the apoptosis of neurons and oligodendrocytes. The final phase of secondary injury, the intermediate-chronic phase, is characterized by cystic cavity formation surrounded by a glial scar of activated astrocytes and extracellular matrix (ECM) proteins. Both features inhibit axonal regeneration, further damaging the SCI lesion [96].

Following traumatic injury and gross neurological examination, the diagnosis of SCI is achieved by imaging. In an acute clinical setting, X-ray and CT scans are the most common imaging techniques for SCI diagnosis. For the evaluation of soft tissue structures such as intervertebral discs, spinal cord, and ligaments, MRI is the optimal imaging method. Treatment for SCI typically involves immobilization of the patient, avoidance of hypotension, administration of neuroprotective drugs (e.g., methylprednisolone sodium succinate), and decompressive surgery [96].

#### Other (ischemic stroke)

Stroke remains a major global health challenge, ranking as the second leading cause of death and the third leading cause of combined death and disability, as measured by DALYs lost worldwide [98]. Acute ischemic stroke, the sudden loss of blood flow to a region of the brain resulting in detrimental neurological function, comprises about 70% of all incident strokes [99]. The predominant cause of ischemic stroke is an embolism, and most of these embolic events are cardioembolic in nature. These cardioemboli typically form as a consequence of underlying heart diseases, where clots that are developed in the heart are dislodged and travel through the bloodstream to the brain, obstructing blood flow to brain tissue. These blood clots obstruct blood flow to areas of the brain, drastically decreasing cerebral blood flow, and causing the pathophysiology of ischemic stroke. During ischemic stroke, cerebral infarction occurs at the primary lesion site, and eventually, loss of electrical function and ROS generation occurs. Moreover, cell membrane function is compromised, and calcium influx occurs, resulting in calcium-dependent excitotoxicity and cell death. When patients experience an ischemic stroke event, the occurrence of irreversible neuronal injury is highly dependent on the severity of the cerebral blood flow drop, as well as the duration of the loss of blood flow [100]. For these reasons, it is crucial to diagnose ischemic stroke promptly and begin treatment. In the clinical setting, ischemic stroke is typically diagnosed via imaging, such as non-contrast CT or MRI. Treatment of ischemic stroke involves restoring blood flow to at-risk tissues (reperfusion therapy) and is most commonly intravenous thrombolysis or endovascular thrombectomy [100].

### Challenges of treating neurological disorders and limitations of current methods

Neurological disorders, namely neurodegeneration and acute CNS injury, are overwhelmingly difficult maladies to treat and cure [101]. The complex and dynamic nature of disease pathophysiology and the unique anatomy of the CNS, inhibit effective therapies. When it comes to pharmacological drugs, a major impediment to effective treatment is the BBB [102]. The BBB is a physical barrier that separates the CNS from the circulating blood and possesses unique properties for the CNS microvasculature [103]. The BBB consists of endothelial cells lining the lumen of the vessel, creating the vessel wall [104]. These endothelial cells differ from those found elsewhere in the body because they are bound together by tight junctions, substantially limiting paracellular transport. The endothelial cells are surrounded by pericytes, substances of the ECM comprising the basement membrane, and astrocyte processes [105]. Due to the unique structure of the BBB, only small lipophilic molecules (molecular weights less than 500 Da) can diffuse across the BBB into the CNS [106]. This requirement significantly restricts the effectiveness of therapeutics in reaching the brain, posing significant challenges in developing pharmacological drugs for CNS treatment.

Because small molecule drugs have a very limited ability to effectively treat CNS disorders, there has been a significant shift towards alternative approaches. Neuronal damage and cell loss in the CNS are fundamental factors that underlie all CNS disorders. Many researchers have invested considerable effort into exploring regenerative cell therapy, also known as stem cell therapy, to restore neuron loss during neurodegeneration and CNS injury [107]. Stem cells from various origins have been investigated for stem cell therapy. Since neurons are the main cell type lost during CNS injury, the foremost stem cell type initially thought appropriate for CNS disorder therapy is neural stem cells (NSCs) [108]. NSCs persist in the subventricular zone (SVZ) of the brain into adulthood and possess minimal regenerative abilities, giving rise to the main CNS cell types: neurons, astrocytes, and oligodendrocytes [108]. Despite the potential for NSCs to replace lost neurons, several limitations challenge their clinical use for CNS disorder treatment. First, NSCs may have to be obtained by harvesting cells from nervous tissue. Their insufficient amount in the human body would not make this method feasible for patients [109]. Alternatively, NSCs could be differentiated from induced pluripotent stem cells (iPSCs). However, the expensive, multistep, laborious differentiation protocol is not suitable for large-scale, uniform generation of NSCs [110]. NSCs transplanted into the brain often cluster near the injection site, showing a limited ability to efficiently migrate toward injury or disease-affected regions. This behavior highlights a key challenge in leveraging the therapeutic potential of NSCs for brain repair and regeneration. Their effectiveness depends on their capacity to reach and repopulate the damaged areas within the CNS [108].

As a result of the limitations mentioned earlier in relation to NSCs, there has been a marked rise in enthusiasm for employing mesenchymal stem cells (MSCs) in stem cell therapy, specifically focusing on the CNS. MSCs can self-proliferate and differentiate into multiple cell lineages. Moreover, they are available in higher abundance in the body and can be isolated from almost all tissues, including bone marrow, adipose tissue, and the umbilical cord [111]. Additionally, the secretome of MSCs has been shown to reduce inflammation as well as promote neural regeneration by containing vascular endothelial growth factor (VEGF), nerve growth factor (NGF), glia-derived neurotrophic factor (GDNF), and brain-derived neurotrophic factor (BDNF) [111]. Although MSCs have many attributes that make them attractive in CNS stem cell therapy, various limitations have affected their clinical utility. During in vitro cell culture, MSCs have been shown to lose their ability to proliferate and differentiate gradually [112]. Furthermore, the in vivo transplantation of MSCs has been implicated in tumorigenesis and cancer progression, thus threatening the use of MSCs in the brain for CNS disorder treatment [111]. Last, a factor that challenges stem cell therapy in treating CNS disorders regardless of stem cell type is the inflammatory and toxic microenvironment of the injury site [111]. This aggressive microenvironment makes stem cell survival post-transplantation very low, thus reducing therapeutic impact. Undeniably, the multifactorial challenges in treating CNS disorders complicate the development of effective treatment options and therapeutics. To overcome the above difficulties, new technologies that permit BBB passage and promote neuronal regeneration must be implemented.

## EVs in the CNS

The intricate and diverse nature of neurological disorders poses significant challenges to existing strategies for treatment and diagnosis within the CNS. These conditions, with their varied origins, complex pathologies, and the CNS’s protective barrier, necessitate novel methodologies for their effective management. In this context, EVs have surfaced as a promising avenue in the field of theranostics [50, 113–117]. These naturally occurring nanoscale entities can cross the BBB and have the inherent capability to transport a wide array of therapeutic and diagnostic agents [118]. EVs suggest a revolutionary shift in addressing the critical demands of therapy and monitoring for CNS diseases [119]. By facilitating the delivery of diagnostic and therapeutic agents across the BBB, EVs help to bridge the gap between the multifaceted nature of neurological disorders and the goal of achieving precise, effective medical interventions in neurological disorders. The transition to EV-based strategies presents a new and exciting prospect in the targeted management of CNS conditions, offering the promise of precise and impactful medical solutions.

### Biogenesis and uptake mechanism of EVs

EVs represent a class of bilayer lipid membranous vesicles secreted by virtually all cell types, including not only healthy cells but also diseased cells, stem cells, and transformed cells [37, 38]. In many prior research into EVs, EVs have been conventionally classified into three main categories: exosomes, microvesicles, and apoptotic bodies based on their biogenesis mechanism [39, 40]. Each type of EV is formed through specific cellular processes, leading to their release into the extracellular space. Exosomes originate from the endosomal pathway and are released by fusion of multivesicular bodies (MVBs) and plasma membranes, with sizes ranging from 30 to 150 nm [120, 121]. Microvesicles are directly formed by outward budding from the plasma membrane, with typical sizes ranging from 100 to 1000 nm. Apoptotic bodies are generated during cell apoptosis with the process of plasma membrane budding and fragmentation [122]. Apoptotic bodies usually have a larger size than both exosomes and microvesicles, with diameters ranging from 800 to 5000 nm [123]. Despite the distinct categories, isolating EVs according to their biogenesis pathway by using current isolation techniques remains a formidable challenge. The challenge arises from the shared biomarkers and overlaps in size among the various EV subtypes [124]. The International Society of EVs has released “Minimal Information for Studies of Extracellular Vesicles 2018 (MISEV2018)” that discourages the use of terms like “exosomes” and “microvesicles”, which imply endosomal origin and plasma membrane, respectively, unless the subcellular origin of the isolated EVs could be validated [125]. In such instances, MISEV2018 has advised the use of “operational terms” to name the isolated EVs with descriptions of isolation method, such as distinct sizes (small EVs (sEVs), less than 200 nm), medium and large EVs (m/lEVs, larger than 200 nm)), specific proteins/cargos/compositions and cellular origins [125]. It is known that exosomes are a subpopulation of EVs distinguished by their pathway of biogenesis and characteristic size distribution. Despite the ongoing debate on EV nomenclature, in this review paper, we will use the terms “sEVs” and “exosomes” interchangeably as they are synonymous. Therefore, advancements in EV isolation and analysis techniques and a deeper understanding of EV biology are needed to refine our ability to distinguish EV subtypes and further harness the potential of EVs in theranostics.

The biogenesis pathway of EVs involves intricate interactions with intracellular vesicles and other organelles, facilitating the incorporation of diverse constituents from parental cells. The enclosure of the molecular components in EVs is mainly mediated by the Endosomal Sorting Complex Required for Transport (ESCRT)-dependent and ESCRT-independent pathways [40]. During the cargo sorting process, EVs may include DNA, RNA, lipids, proteins, and metabolites, among other molecular components. sEVs have been identified to be enriched with protein markers that play crucial roles in their biogenesis, release, and functionality. These markers contribute to the identification and characterization of these vesicles. Among the key proteins found in sEVs are tetraspanins (CD63, CD9, and CD81), membrane trafficking proteins (RAB proteins and annexins), and proteins involved in MVBs formation (ALIX, TSG101, and clathrin) [40]. Therefore, EVs play a role as carriers that can mimic, to some extent, the intracellular, physiological, and pathological conditions of their parental cells. In the realm of disease diagnosis and prognosis, the inherent capacity of EVs to encapsulate a spectrum of molecular cargo from parental cells positions them as promising liquid biopsy makers across various diseases, such as cancers, neurological diseases, and cardiovascular diseases [113]. Upon releasing into the extracellular space, EVs can enter various biofluids, including blood, urine, CSF, milk, and saliva, making them accessible targets for non-invasive diagnostic approaches [126].

### Function of EVs as intercellular communicators in the CNS

In the CNS, the primary focus of neuronal communication has been on synaptic communication, where neurotransmitters are released from synaptic vesicles in the presynaptic neuron and bind to receptors on the postsynaptic neuron [41]. This process is crucial for transmitting signals across the synaptic cleft and is fundamental to brain function. Recent research has identified EVs as another crucial mechanism for intercellular communication in the brain [42]. Unlike synaptic vesicles, which are limited to synapses, EVs can facilitate communication over longer distances [43, 44].

**Fig. 2:**
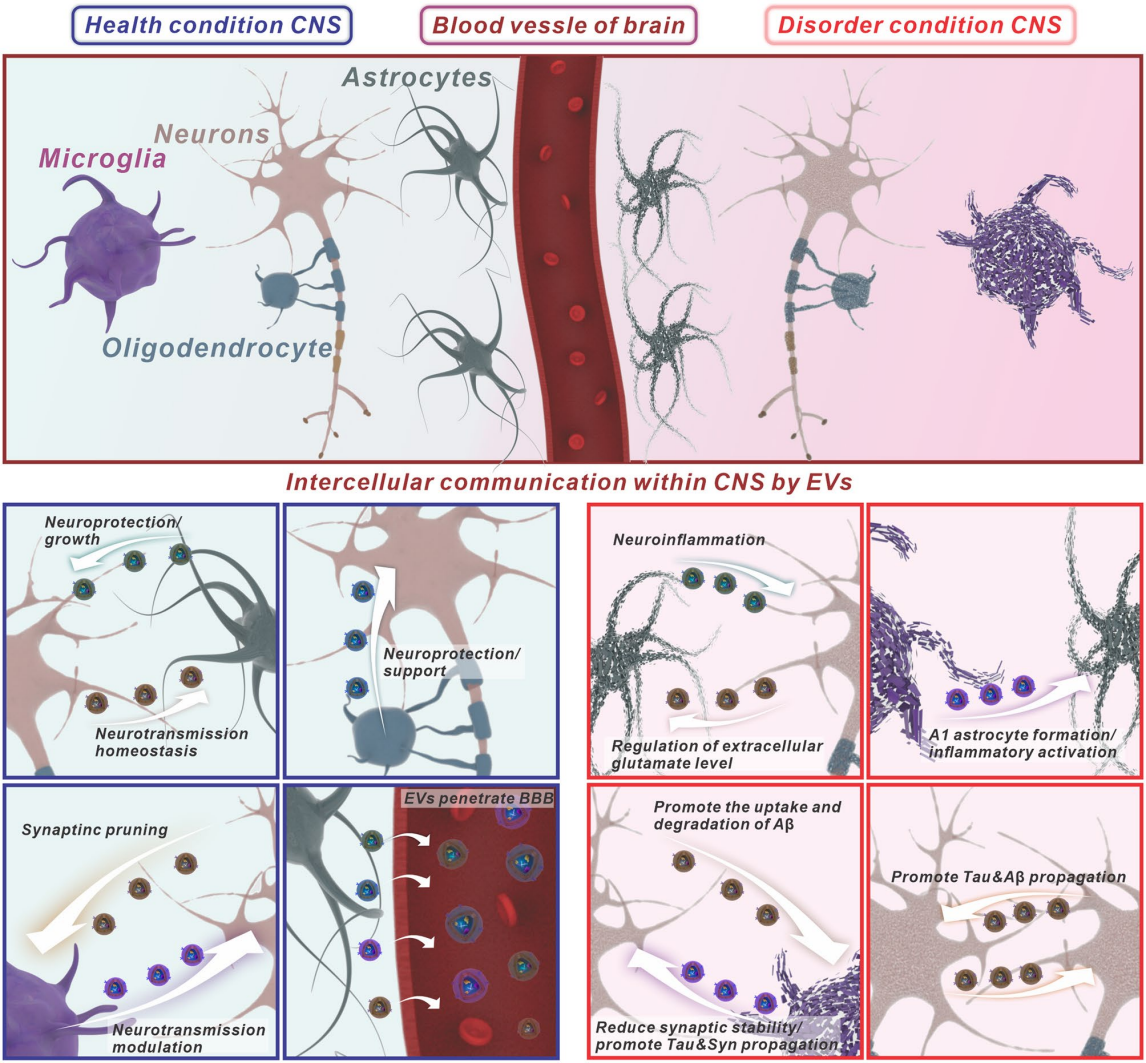
EVs-mediated intercellular communication in the CNS. The interplay of EVs with neural cells highlights their significance in both normal CNS function and pathological conditions. Under normal circumstances, EVs contribute to CNS homeostasis by supporting neuronal health, ensuring myelination, enhancing synaptic plasticity, offering neuroprotection, and providing trophic support to neurons. Conversely, in disease states, EVs originating from compromised CNS, such as dysfunctional neurons, activated microglias and reactive astrocytes cells, can propagate harmful misfolding proteins or inflammatory factors to recipient cells. This activity facilitates the progression of neurodegenerative diseases and exacerbates neuroinflammation and neural damage. Furthermore, EVs derived from CNS cells can cross the BBB and be identified in the bloodstream. The specific cargoes of these EVs can serve as biomarkers, reflecting the pathological state of their cells of origin and correlating with the stages of neurological disorders

EVs serve as potent mediators of intercellular communication by transferring information between cells, including neurons, astrocytes, oligodendrocytes, and microglia [42, 127, 128] [Fig. 2]. EVs can be internalized by recipient cells through various mechanisms, such as receptor-mediated interactions, endocytosis, and membrane fusion [45]. Through these processes, EVs deliver a payload of proteins, mRNAs, and non-coding RNAs to acceptor cells. The transferred molecular components with varying bioactivities have the potential to induce functional effects on recipient cells [45]. The interplay of EVs with parental and recipient cells highlights their significance in both normal CNS function and pathological conditions [45]. Accumulating evidence supports that EVs play important functions in neuronal homeostasis maintenance within the CNS, including maintenance of myelination, modulation of synaptic plasticity, antigen presentation, and trophic support of neurons [48]. Conversely, EVs from dysfunctional CNS cells may exert detrimental effects by propagating misfolded proteins or inflammatory factors to the neighboring cells, contributing to the spread of neurodegenerative processes or exaggerating neuroinflammation and neurodamage [45]. Understanding the dual roles of EVs in maintaining neuronal homeostasis and contributing to neurodegenerative processes is crucial for developing early diagnosis methods and targeted therapeutic strategies [49]. Research in this area aims to harness the beneficial functions of EVs while mitigating their detrimental effects to develop interventions for neurological disorders. Understanding the molecular composition of EVs, biogenesis, and uptake mechanisms is critical for harnessing their diagnostic and therapeutic potential, as the cargo they carry may influence their biological effects on recipient cells in various physiological and pathological contexts. As the field progresses, insights into the specific cargo and mechanisms of EV-mediated communication within the CNS will likely uncover novel nanotheranostics avenues for neurological diseases.

### Role of EVs in neurological disorders

#### Neurodegenerative diseases

Neurodegenerative diseases, such as AD characterized by Aβ and tau accumulation, PD associated with α-synuclein aggregates, and ALS marked by superoxide dismutase 1 (SOD 1) misfolding, are increasingly understood in the context of sEVs involvement [45, 113, 129]. Recent research has highlighted the significant role of sEVs in the propagation of these misfolded proteins, suggesting they may facilitate intercellular transfer and contribute to the progression and severity of these conditions [130–132]. AD is characterized by neuropathological hallmarks, including extracellular plaque deposits of Aβ protein and NFTs composed of insoluble p-Tau aggregates [133, 134]. EVs released from brain cells affected by AD, such as neural cells that undergo degeneration, have been identified to carry pathological proteins associated with the disease [128, 135]. Several studies have shown that both extracellular free Aβ and Aβ-contained EVs play a role in the progression of AD pathology [136–140]. A previous study has shown that sEVs isolated from the brains of AD patients contain elevated levels of Aβ oligomers [141]. These EVs have been discovered as possible transporters for transferring toxic protein species from neuron to neuron, as has been shown in vitro. This finding suggests a novel mechanism by which Aβ oligomers might propagate through sEVs within neural networks, contributing to the disease’s progression. The content and function of EVs can differ significantly depending on their cell of origin, reflecting the cellular context and state, especially under disease conditions [40, 142]. In AD, EVs released from brain cells, particularly microglia and astrocytes, have been implicated in exacerbating neuroinflammation, a critical component of AD pathology [137, 143]. A study by Asai and colleagues demonstrated that exosomes derived from microglia can facilitate the spread of tau protein, another critical pathological hallmark of AD and other tauopathies [144]. This finding indicates that exosomes can also contribute to the progression of neurodegenerative diseases by spreading pathogenic proteins. The study also showed that inhibiting exosome synthesis significantly reduced tau propagation *in vitro and in vivo*. This suggests a potential therapeutic strategy to limit disease progression by targeting exosome-mediated mechanisms. EVs may also carry pro-inflammatory molecules, contributing to an inflammatory environment that accelerates neuronal damage. However, the exact function of EVs in AD biology is still unclear and under investigation. This discovery provides potential avenues for early detection and monitoring of AD.

In PD, the pathological accumulation of α-syn, a key protein, begins in the brain years before the clinical symptoms manifest. Although elevated concentrations of α-syn in blood have been proposed as an early biomarker of Parkinson’s disease, identifying brain-derived α-syn is challenging due to its ubiquitous presence in different tissues, such as CSF, blood, saliva, tears, and peripheral tissues [145, 146]. Addressing this challenge, a study isolated neuronal exosomes from the blood plasma of participants, including 36 patients with early-stage PD, 17 patients with advanced PD, 20 patients with idiopathic rapid eye movement sleep behavior disorder, and 21 healthy controls [147]. The study revealed that levels of exosomal α-syn were significantly higher in individuals with PD compared to controls. Furthermore, these levels correlated with the severity of motor and non-motor symptoms in PD patients. This suggests that exosomal α-syn in blood plasma could serve as a specific biomarker for early PD progression detection and monitoring, offering a novel approach to understanding and managing this neurodegenerative disease. Another research conducted by Guo et al. showed that microglial exosomes contribute to the progression of α-syn pathology [148]. A notable aspect of their research was that they could apply their findings to a clinical setting. They achieved this by isolating exosomes derived from microglia/macrophages in the CSF of PD patients. In doing so, they successfully detected the presence of α-syn oligomer in CD11b + exosomes. By inducing α-syn aggregation in neurons, the observed exosomes in this study provide strong evidence for the translational relevance of these findings.

Several studies have predominantly focused on the neurotoxic proteins contained within EVs; however, there is a growing amount of research investigating abnormally upregulated and downregulated microRNAs (miRNAs) in neurodegenerative diseases [149–151]. miRNAs are small, non-coding RNA molecules that play a critical role in regulating gene expression. Abnormal alteration in miRNA expression can influence the pathways that lead to neurodegenerative conditions, such as AD, PD, and other neurodegenerative diseases [152]. Brain-derived EVs have been found to harbor the ability to penetrate the BBB. This breakdown is crucial as it potentially allows for releasing these EVs into peripheral circulation [54–57]. The unique profiles of these biomolecules in EVs provide valuable insight, offering a promising avenue for the development of biomarkers for neurodegenerative diseases. One of the most promising aspects of miRNA research in neurodegenerative diseases is their potential as biomarkers that could aid in early diagnosis and monitoring of disease progression [45, 153]. Since miRNAs-contained EVs are stable in bodily fluids and their expression profiles can be disease-specific, they are promising candidates for developing non-invasive diagnostic tools. Cheng et al. profiled serum exosomal miRNAs to differentiate between healthy individuals and patients with AD [154]. Utilizing unbiased next-generation deep sequencing followed by quantitative reverse transcription polymerase chain reaction (qRT-PCR) for validation, they identified a distinctive AD-specific signature comprised of 16 miRNAs. This signature demonstrated high predictive accuracy, with a sensitivity of 87% and specificity of 77% for AD diagnosis. Gui et al. developed a novel miRNA profiling strategy to analyze exosomal miRNAs isolated from CSF of patients with PD and AD [155]. Their findings indicated a significant alteration in the exosomal miRNA landscape: sixteen miRNAs were upregulated, while eleven were downregulated in PD CSF compared to healthy controls. Notably, miRNA-1 and miRNA-19b-3p were further validated and found to be significantly reduced in independent PD samples. On the contrary, miRNA-153, miRNA-409-3p, miRNA-10a-5p, and let-7 g-3p showed significant overexpression in PD CSF exosomes. Similarly, many studies have been done to investigate exosomal miRNA expression patterns associated with neurodegenerative diseases [156–158]. The findings of this study are significant, not only in advancing the understanding of AD at the molecular level but also in potentially offering a novel, non-invasive biomarker approach for early and more accurate diagnosis of AD.

MiRNAs within EVs play a pivotal role in facilitating complex communication networks between neurons, astrocytes, glial cells, and vascular cells in the brain [127, 159]. This intercellular exchange of miRNAs can regulate gene expression across different cell types, influencing brain development, function, and the response to injury or disease. This could include modulation of neurotoxicity [160], inflammation [161, 162], and immunoactivity [163, 164]. Understanding the roles of specific miRNAs in neurodegenerative diseases holds significant promise for novel therapeutic strategies as well. Targeting distinct miRNAs within EVs may offer a unique approach to modulate key disease-related pathways. This strategy could involve either inhibiting miRNAs that contribute to disease progression or enhancing the function of beneficial miRNAs. miR-124, one of the most abundant miRNAs in the CNS, has been implicated in various pathologies within this system [165]. Its dysregulation is closely related to the development and progression of neurodegenerative diseases [166]. Recent therapeutic approaches involving the delivery of miRNA-124 have demonstrated a neuroprotective role, showing promise in treating neurodegenerative disorders and brain traumas [167, 168]. Ge et al. discovered that miR-124 is abundantly present in sEVs derived from M2-microglia. When administered intravenously, these exosomes were found to inhibit neuronal inflammation and autophagy, enhance neurogenesis, and consequently improve neurological outcomes in mice [169]. Specifically, in mice models of repetitive mild TBI, these microglial exosomes were observed to be internalized by neurons in the injured brain. Notably, miR-124-3p within these exosomes was transferred to hippocampal neurons, where it mitigated neurodegeneration by targeting the Rela/ApoE signaling pathway. Furthermore, the potential of EVs as delivery systems for therapeutically delivering siRNA and miRNAs presents an exciting opportunity [170–173]. A new finding has demonstrated that EVs can efficiently cross the BBB, unlocking transformative potential in the treatment of neurodegenerative diseases. Engineered EVs can be specifically tailored to transport therapeutic miRNAs directly to targeted cells within the brain. This precision delivery system not only overcomes the significant challenge of BBB penetration but also promises a new level of specificity in treating brain disorders, potentially revolutionizing our approach to neurodegenerative disease therapy. The field of miRNA research in neurodegenerative diseases, particularly within EVs, is still in its infancy. Challenges include identifying disease-specific miRNA signatures, understanding the mechanisms of miRNA loading into EVs, and elucidating the exact role of these miRNAs in disease progression [174]. Further research in this area is crucial for unlocking the full potential of miRNAs in the diagnosis and treatment of neurological disorders.

#### CNS injuries

EVs have gained significant attention in CNS injuries, specifically traumatic brain injuries (TBI) and spinal cord injuries (SCI), due to their intricate involvement in injury response, repair processes, and their potential applications as therapeutic agents and biomarkers [175–177]. CNS-derived EVs are rich in cell-specific surface markers and contain diverse intraluminal contents, including proteins, RNA, DNA, and metabolites. By studying these components, we can better understand the biochemical and molecular conditions of neurons and glial cells during neurologic injury. Moreover, these insights provide valuable information about the intrinsic repair mechanisms of the CNS [178]. The evolution of EV isolation techniques, when integrated with the analysis of comprehensive biomarker profiles and clinical data, holds promise for enhancing the classification and treatment of CNS injuries and their long-term consequences.

With TBI, the ability of EVs to traverse the BBB is particularly advantageous, offering novel diagnostic strategies and therapeutic avenues [179]. The molecular cargo of EVs, especially those released from injured brain cells, can modulate the immune response within the CNS, potentially influencing the trajectory of injury and subsequent recovery processes. By carrying molecules with pro-inflammatory or anti-inflammatory actions, EVs can either exacerbate or mitigate secondary injury mechanisms, thereby affecting the progression of neuronal damage and the development of cognitive deficits post-TBI [180]. The utilization of liquid biopsy as a minimally invasive alternative to traditional imaging and behavioral assessments facilitates the ongoing monitoring of biochemical and molecular alterations in the CNS [181–183]. The targeted analysis of specific EV cargos, derived from various brain cell types, could refine our understanding of TBI endophenotypes, including neuroinflammation, axonal injury, and neurodegeneration. Efforts to identify EV-based biomarkers for TBI have largely centered on well-characterized proteins like UCHL1, Tau, and Aβ [184–186]. Recent findings of elevated p-Tau within neuronally enriched EVs in TBI subjects highlight the dynamic processes and time-dependent mechanisms at play in TBI [185, 187, 188]. These biomarkers, along with indicators of neuroinflammation, may shed light on the factors contributing to TBI’s progression to AD and the biological response to therapeutic interventions. Growing experimental evidence suggests that EVs are potentially useful in therapeutic interventions [189–191].

Like TBI, EVs in SCI can influence the immune environment at the injury site. EVs have functional roles in neuron–glia and CNS–periphery communication following neurotrauma by regulating neuronal function, axonal regeneration and remyelination, metabolic activity, and the inflammatory milieu [192, 193]. Understanding EV signaling after SCI is limited, but recent studies have identified differences in circulating EV counts and miRNA cargoes that can contribute to remote inflammatory changes. Following SCI, astrocytes in the CNS undergo a series of changes collectively referred to as reactive astrogliosis [194, 195]. The release of EVs by reactive astrocytes serves as a means of communication with other glial cells, neurons, and even cells of the immune system [193, 196]. These EVs, originating from reactive astrocytes, are found to be rich in certain small GTPases, such as profilin-1 and fascin actin-bundling protein 1, as well as proteins like IL-1β and HIV-1 protein Nef, and miRNAs [197]. These molecules can impede neuronal functions by reducing neurite extension, dampening spike firing rates, and potentially leading to neuronal cell death. Furthermore, in the context of TNF-α or IL-1β stimulation, astrocyte-derived EVs have been shown to contain miR-125a-5p and miR-16-5p [198]. These miRNAs are implicated in the downregulation of the neurotrophin receptor NTRK3 in neurons, which subsequently diminishes dendritic complexity and synaptic activity.

EVs have emerged as pivotal elements in both the pathophysiological progression and recuperative processes associated with TBI and SCI. EVs can significantly modulate inflammation, drive neurodegeneration, and facilitate tissue repair. As a result, they present remarkable opportunities for creating innovative therapeutic strategies and discovering new biomarkers. Their involvement in these crucial biological processes puts them at the forefront of potential advancements in medical treatment and diagnostics. The continued exploration of EVs is essential to fully understand their diverse functions and to harness this knowledge for therapeutic innovation. Progress in this field holds the potential to transform the landscape of clinical treatment for TBI and SCI, enhancing patient outcomes through targeted interventions that are informed by a deep molecular insight.

## Applications of EVs as nanotheranostic targets in CNS

Nanotheranostics represents a cutting-edge convergence of nanotechnology, diagnostics, and therapeutics [199–201]. The nanotheranostic potential of EVs in CNS diseases is an exciting and rapidly developing area of research that explores the use of EVs for both therapeutic and diagnostic purposes in the CNS [49–51]. EVs present a natural and versatile option for this purpose. Because of the endogeneity of EVs, EV-based theranostics possess inherent biocompatibility and minimal immunogenicity compared to synthetic nanomaterials [202–205]. Their ability to cross the BBB and deliver a wide range of cargo, including proteins, lipids, nucleic acids, and drugs, makes them ideal for targeting CNS pathologies. Certain EVs may carry molecules with inherent therapeutic effects, potentially providing a dual function of acting as both delivery vehicles and therapeutic agents.

The utilization of EVs as therapeutic delivery vehicles within the CNS has mainly concentrated on two distinct strategies: first, leveraging native EVs that inherently possess therapeutic factors, and second, custom-engineering EVs to encapsulate and transport specific therapeutic agents. These approaches capitalize on the natural ability of EVs to cross biological barriers and deliver their cargo directly to target cells, offering a promising avenue for the development of novel CNS-targeted therapies [113, 206]. A growing body of research has illuminated the therapeutic potential of EVs derived from a diverse array of cell types [207, 208]. This includes MSCs [209–211], iPSCs [212], brain-derived NSCs [213], embryonic stem cells (ESCs) [214], and dendritic cells (DC) [215]. Among these, stem cell-sourced EVs are widely used for their intrinsic therapeutic properties and versatility as drug and gene delivery vehicles. Both native and bio-engineered forms of these stem cell-derived EVs offer an interesting modality for the development of novel treatment paradigms for a spectrum of brain diseases. EVs sourced from immune cells, such as DCs, macrophages, and natural killer (NK) cells, demonstrate an increased ability to evade immune clearance [38]. This attribute allows them to remain longer in the peripheral circulation, potentially leading to improved therapeutic efficacy. By exploiting the unique capabilities of these EVs to encapsulate and transport bioactive molecules across the BBB, researchers are paving the way for breakthrough therapies that may offer hope for conditions currently deemed intractable.

Great strides have been made in the field of EV engineering to tailor EV cargoes or surface membranes for enhanced delivery or targeting. The most common methods for EV engineering can typically be divided into the engineering of the parent cell for modified EV release or direct engineering of the EVs. Various cargoes can be indirectly loaded into EVs by engineering the parent cells through co-incubation or gene transfection [216]. Small molecule drugs, such as curcumin and paclitaxel, can be co-incubated with parent cells at various conditions for loading into EVs [217, 218]. Transfection of the parent cells with exogenous nucleic acids (DNA plasmids, siRNAs, miRNAs, etc.) is a common method for loading nucleic acid cargoes into EVs [219]. Direct loading of the EVs with specific cargoes can be achieved using methods such as passive incubation, electroporation, or mechanical methods [220]. Like the co-incubation of cargoes with parent cells, hydrophobic, small-molecule drugs can be directly loaded into EVs by passive mixing [221]. Moreover, larger cargoes, such as biomolecules, can be successfully introduced directly into EVs through the transient pores formed during electroporation. Other mechanical methods also rely on disrupting the EV membrane for cargo loading, including freeze-thaw cycles, sonication, and extrusion [222]. The aforementioned strategies engineer EVs with functional cargoes to create novel nanotherapeutic vehicles.

Besides modifying the cargoes that are loaded into EVs, there is great interest in modifying the surface membranes of EVs for enhanced cell-specific targeting. Modifying the surface membranes of EVs can be achieved by engineering the parent cells or by directly engineering the EVs. The surface membranes of EVs released from parent cells can be modified by gene engineering of the parent cells via transfection [216]. Transfection of the parent cells can enrich the expression of desired proteins onto the surface of the secreted EVs. In addition to the genetic engineering of parent cells, metabolic engineering by incubating cells with L-aziodohomoalanine or Nazidoacetyl-D-mannosamine can affect new surface compositions on the EVs [223]. Parent cells can also be engineered by fusion with fusogenic liposomes to modify their surface compositions [224]. Liposome fusion is also a method to directly modify EVs [225]. Additionally, direct modification of EV surface membranes can also be achieved through lipid post-insertion or adsorption of substances onto the EV membrane. Another standard method for direct engineering of EV surfaces includes the chemical modification of ligands onto the EV surface [226]. Despite the multitude of strategies for EV engineering, limitations involving loading efficiency and insufficient targeting precision challenge the clinical application of engineered EVs.

While EVs offer promising potential for CNS theranostics, EVs often lack the long-lasting stability, potency, and targeting precision needed for effective disease diagnosis and treatment [227]. Nanomaterials have emerged as a revolutionary tool in drug delivery, offering distinctive advantages such as enhanced loading capacity, robust protection for encapsulated drugs, precise control over drug release kinetics, and improved targeting of specific tissues or cells [52]. These properties stem from their customizable surface chemistry and physical dimensions, which can be tailored to navigate the complex biological environments within the body. Despite these benefits, the application of nanomaterials is hindered by several significant challenges. Non-specific cytotoxicity, arising from unintended interactions with non-target cells, and suboptimal biocompatibility, which can trigger adverse immune responses, are among the primary concerns [53, 228]. Additionally, achieving efficient delivery to the desired site without loss of functionality or integrity remains a formidable challenge. Addressing these issues requires a comprehensive understanding of the interface between nanomaterials and biological systems.

The integration of EVs with nanomaterials represents a frontier in CNS nanotheranostics, offering novel solutions for the challenges of drug delivery, therapeutic efficacy, and disease diagnosis in the brain [229, 230]. As research progresses, the development of these bio-nanomaterials promises to bring about significant advancements in treating and understanding CNS diseases. In this review, we will summarize the latest developments in the growing field of EVs as nanotheranostics agents, highlighting their natural versatility and the integration with nanomaterials to overcome current limitations and amplify therapeutic and diagnostic efficacy in CNS. This section updates the latest trends in EVs as nanotheranostics targets for personalized medicine, organized by applications in different CNS diseases.

### AD

**Fig. 3:**
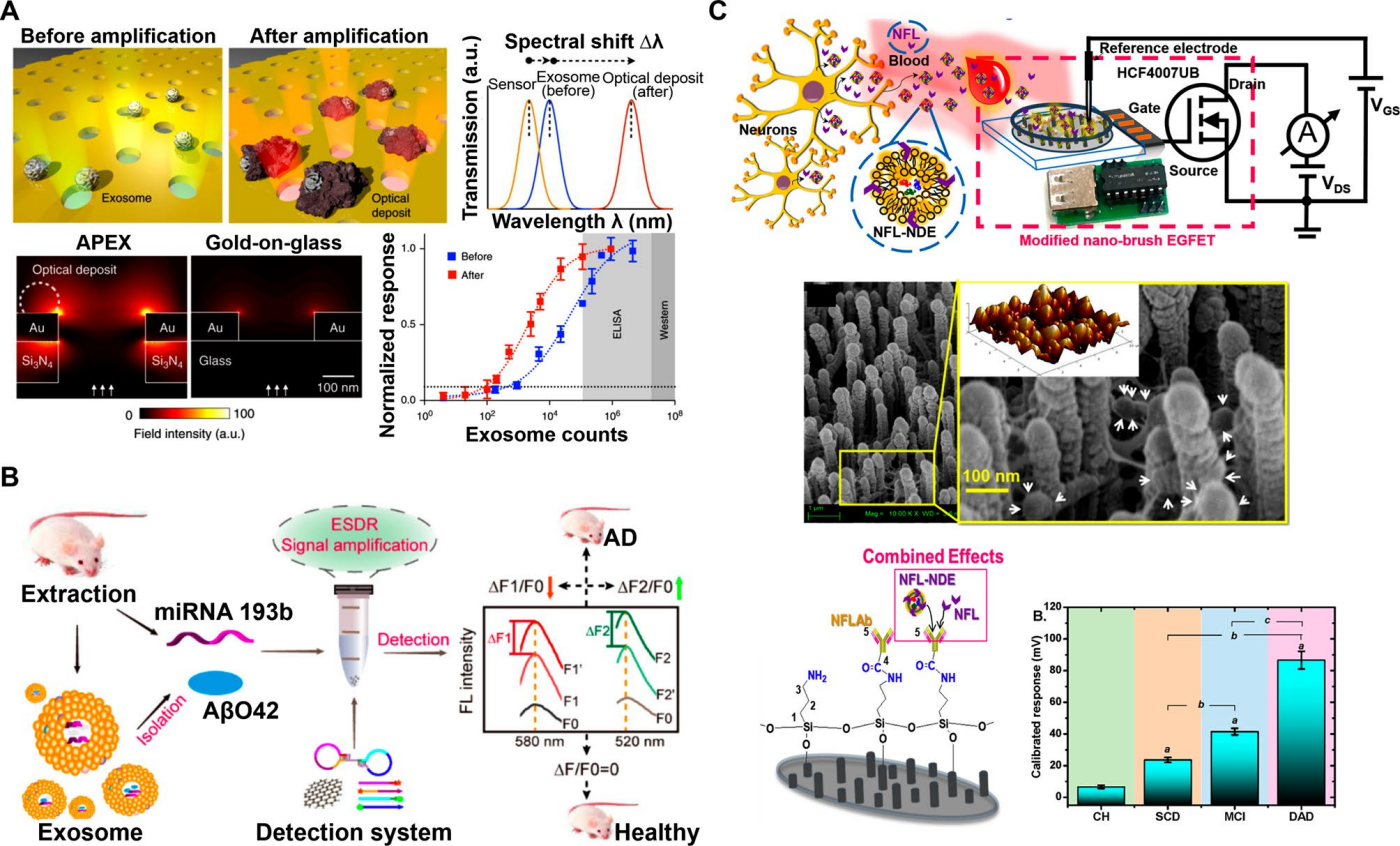
Nanodiagnostic platforms for the detection of AD-derived EVs. **(A)** APEX assay schematics. (Top left), A representative schematic of changes in the transmission spectra with APEX amplification. (Top right), Finite-difference time-domain simulations show enhanced electromagnetic fields with back illumination. (Bottom left), and comparison of the detection sensitivities of APEX, enzyme-linked immunosorbent assay (ELISA), and western blotting. (Bottom right) Reproduced with permission from [232]. Copyright © 2019, The Author(s), **(B)** Schematic diagram of the detection process of the dual recognition of miRNA-193b and AβO42, Reproduced with permission from [236]. Copyright © 2023, American Chemical Society. and **(C)** Sensing scheme of the integrated nano-brush sensing substrate with the EGFET device (Top), SEM images show the presence of NFL-specific NDEs (white arrows), with atomic force microscopy (AFM) scan (inset) showing the surface roughness of post-NFL-sensing on nano-brush sensing substrates (Middle), schematic illustration depicting the important chemical bonds after sequential treatments and detection of NFL substances on the nano-brush sensing substrate (Bottom left), and Statistical analyses of the calibrated responses among the four tested groups (Bottom right). Reproduced with permission from [238]. Copyright © 2023, American Chemical Society

Surface plasmon resonance (SPR) biosensing is an advanced spectroscopic technique that allows for the quantitative measurement of molecular binding events in real-time. This method relies on the excitation of surface plasmons, coherent electron oscillations at the interface between a metal and a dielectric medium, whose resonance is sensitive to changes in the refractive index near the sensor surface [231]. Such changes occur when molecules bind to a functionalized surface, making SPR a powerful tool for monitoring these interactions without the need for labeling. This label-free approach not only preserves the natural state of the interacting analytes but also facilitates sensitive analysis with surface modification. Lim et al. have developed a highly sensitive analytical platform, termed Amplified Plasmonic Exosome (APEX), designed for multiplexed analysis of exosome populations directly from the blood samples of patients with AD [232]. APEX technology uniquely measures different populations of circulating Aβ proteins, distinguishing between exosome-bound and unbound forms. Utilizing a double-layered plasmonic structure consisting of a periodic array of size-matching gold nanoholes over a patterned silicon nitride membrane, APEX achieves enhanced bidirectional SPR measurement, as illustrated in Fig. 3a. This configuration minimizes temperature fluctuations in enzymatic activity, a critical factor in the platform’s performance. The APEX assay, with its superior sensitivity (detecting $\sim$ 200 exosomes), allows for the co-localization of multiple targets. Consequently, the researchers established a distinct population of exosome-bound Aβ (Aβ42 + CD63+) and successfully quantified its presence in blood samples from both AD patients and healthy controls. Notably, the measurement of exosome-bound Aβ yielded a more precise representation of amyloid plaque deposition in the brain, as demonstrated by PET scans, compared to the total levels of circulating or unbound Aβ.

Another plasmonic biosensor was developed by Song et al. to detect exosomal miRNAs from human serum for AD diagnosis [233]. In this study, they created a label-free plasmonic biosensor system designed to detect exosomal miRNAs from human serum for AD diagnosis. This biosensor is constructed based on DNA-assembled advanced plasmonic architecture (DAPA). The designed nanoarchitecture features two narrow nanogaps, which facilitate strong plasmon coupling. This interaction, in turn, enables the plasmonic single-nanoparticle-based sensors to interact with incident light to generate localized SPR (LSPR), substantially boosting the optical energy density and achieving a 1.66-fold enhancement in refractive-index (RI) sensitivity compared to traditional nanorods at LSPR wavelengths. Furthermore, locked nucleic acid (LNA) probes were integrated, known for their high-affinity binding to specific RNA sequences. This addition has propelled the proposed biosensor to ultrasensitive and selective detection of single-nucleotide variants of exosomal miRNAs at the attomolar concentration level. Clinically, the biosensor demonstrated its potential by accurately distinguishing between AD patients and healthy individuals by measuring exosomal miRNA-125b, miRNA-15a, and miRNA-361 levels in serum samples. Notably, a combination of exosomal miRNA-125b and miRNA-361 revealed the most robust diagnostic performance, exhibiting a sensitivity of 91.67%, a selectivity of 95.00%, and an overall accuracy of 99.52%. These results underscore the biosensor’s significant promise as a blood-based diagnostic tool to profile AD-associated exosomal miRNAs. Building on their initial work, Song et al. have pioneered a second-generation biosensor with a programmable curved plasmonic nanoarchitecture, specifically designed for the precise profiling of exosomal miRNAs in the clinical diagnosis of AD [234]. The innovation of this biosensor lies in its pre-designed nanoarchitecture, which, through computational simulation, introduces a tailored nanoscale space that generates a potent electromagnetic field [235]. This field is crucial for enhancing molecular interactions and signal detection. The nanoarchitecture is actualized using DNA-directed crystallization technology, enabling precise control over the bending angles of the structure. By manipulating the structures at the sub-nanometer level, biosensors have shown their clinical utility by successfully classifying individuals into categories of AD, mild cognitive impairment (MCI), and healthy controls.

Due to the complex and heterogeneous nature of AD, it is significantly challenging to diagnose AD using a single biomarker. A multiplexed approach, which combines two or more biomarkers, can greatly enhance the specificity and sensitivity of AD diagnostics. Recently, Zhou and colleagues have advanced the field of diagnostics with the development of a highly sensitive and non-invasive fluorescent biosensor for simultaneously detecting two critical biomarkers associated with the early stages of AD [236], as illustrated in Fig. 3b. This innovative approach can detect neurogenic exosomes-derived miRNA-193b and Aβ42 oligomers (AβO42) on the same platform, both indicative of the pathophysiological alterations characteristic of early AD. The biosensor features an innovative enzyme-free signal amplification mechanism. This system employs an entropy-driven strand displacement reaction (ESDR), a process that facilitates the amplification of the detection signal with no enzymatic intervention. The assay operates by specific binding of miR-193b and AβO42 to their respective aptamers. Graphene oxide (GO) as a nano component is integral to this system to diminish background fluorescence and enhance overall sensitivity. The detection limits achieved for miR-193b and AβO42 were 77 pM and 53 pM, respectively. The sensor’s ability to concurrently identify an increase in AβO42 and a decrease in miR-193b within exosome samples enables the differentiation of early-stage AD from non-AD controls.

To detect trace AD biomarkers in complex biological samples, bioelectronics, especially transistor sensors, which feature exceptional capacity to transduce and amplify biological and biochemical inputs into electronic signals, facilitate the detection and analysis of complex biological events. Li and coworkers developed a graphene electrolyte-gated transistor biosensor for detecting serum neuron-derived exosomal Aβ42 (NDE-Aβ42), a biomarker rapidly gaining recognition for its relevance in AD [237]. In this work, a nanobiosensor-based platform is constructed by a AuNP-decorated graphene electrolyte-gated Field-Effect Transistor (G-EGT). This biosensor’s design incorporates an advanced antifouling strategy, utilizing a dual-blocking process that effectively reduces interference from the complex biological matrix of serum, enhancing detection sensitivity. With a limit of detection at the attogram level (447 ag/mL). The assay’s performance was validated against the SH-SY5Y-derived exosomal Aβ42, yielding results highly consistent with ELISA results. Crucially, clinical analysis conducted on a cohort of 27 subjects underscored the superior diagnostic value of measuring NDE-Aβ42 over conventional serum Aβ42. This biosensor is the first electronic test that detects NDE-Aβ42 with great sensitivity and specificity. Its clinical relevance was further proved by its capacity to reliably distinguish between AD patients and non-AD persons and differentiate AD from vascular dementia, with an unparalleled accuracy of 100% and a Youden index of one.

The levels of neurofilament light chain (NFL) and neuron-derived exosomes (NDEs) in human blood plasma/serum are being studied as potential biomarkers for early detection of AD. The identification of AD-related NDEs, especially those containing NFL, could help improve early diagnosis of AD. Lin et al. have developed a new transistor sensor to measure NFL-specific NDEs in human blood plasma/serum [238]. In this study, a nano-brush structure, composed of vertically aligned High-aspect-ratio upstanding silicon nanowires (USNW) coated with indium tin oxide, serves as a highly effective sensing substrate, as seen in Fig. 3c. This structure is bio-functionalized with an NFL-specific antibody (NFLAb) through silane coupling chemistry, enabling the selective capture of NFL-containing NDEs, and further connected as the extended gate in a field-effect transistor (EGFET). The team has demonstrated the sensor’s ability to detect NFL-containing NDEs with a high level of sensitivity, achieving a limit of detection (LOD) of 60 NDE/mL and a limit of quantification (LOQ) of 6 × 10^3^ NDE/mL.

Although native stem cell-derived EVs exhibit promising therapeutic properties, their innate inability to specifically recognize and target diseased neuronal cells poses a significant challenge. To address this, research is now pivoting towards developing engineered EVs with enhanced targeting capabilities [226, 239, 240]. By incorporating target delivery elements into EVs, scientists aim to confer the ability to discriminate between healthy and diseased cells within the brain. These modifications guide the EVs directly to the affected neuronal populations, improving the specificity and efficacy of EV delivery of therapeutic agents.

**Fig. 4:**
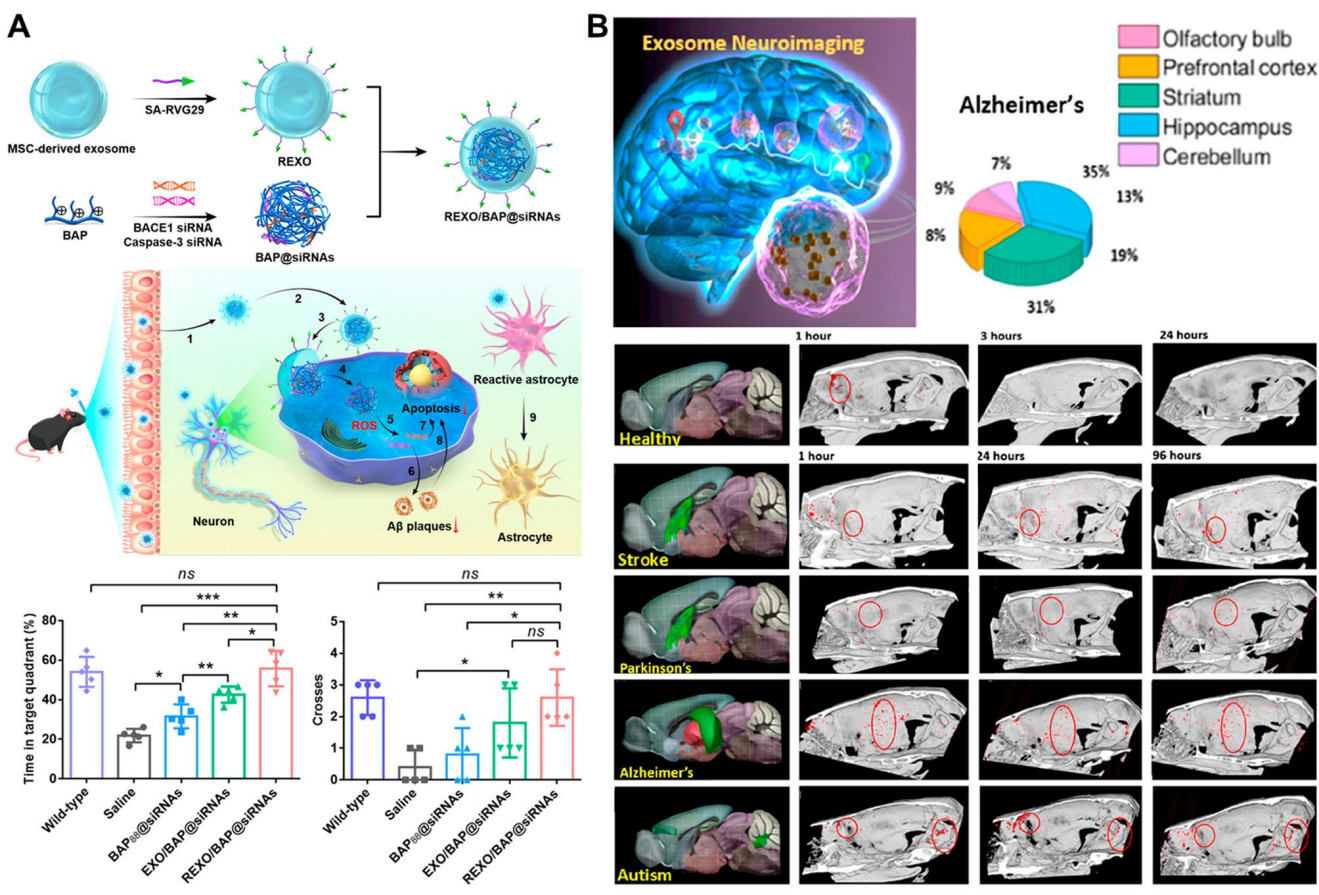
EV-based nanotherapeutic platforms for AD therapy. **(A)** Schematic diagram of the Lesion-Recognizing NPs and mechanism of the Lesion-Recognizing NPs for the synergistic treatment of AD (Top), Behavior improvement of the experimental mice. Time spent by mice in the target quadrant (Bottom left), Number of times the mice crossed the hidden platform (Bottom right). Reproduced with permission from [242]. Copyright © 2023, American Chemical Society, and **(B)** Schematic diagram of the gold nanoparticle-labeled exosome neuroimaging in AD (Top), and longitudinal homing and accumulation of MSC-exo in diseased brains (Bottom). Reproduced with permission from [244]. Copyright © 2019, American Chemical Society

Alvarez-Erviti et al. at Oxford University have pioneered a novel approach employing exosomes as a delivery system for short-interfering RNA (siRNA) to the brain in mice [241]. To minimize immunogenicity, the team utilized self-derived DCs to generate exosomes. These DCs were genetically engineered to express the exosomal membrane protein Lamp2b, fused to the neuron-specific rabies virus glycoprotein (RVG) peptide to achieve targeted delivery. The resulting purified exosomes were then loaded with therapeutic siRNA molecules via electroporation. Upon intravenous injection, these RVG-targeted exosomes demonstrated a remarkable capacity to deliver GAPDH siRNA specifically to neurons, microglia, and oligodendrocytes within the brain, effectuating a targeted gene knockdown. This research reached a pivotal milestone in demonstrating the therapeutic potential of this exosome-mediated siRNA delivery strategy by achieving a significant knockdown of both mRNA (60%) and protein (62%) levels of BACE1, which is a crucial target implicated in AD in wild-type mice. Recently, Li et al. advanced targeted drug delivery by modifying MSC-derived exosomes (MSC-exo) with RVG peptide with 29 amino acids (REXO), which binds specifically to neuronal acetylcholine receptors [242]. They further complexed therapeutic siRNAs with a ROS-responsive polymer, cationic polymer poly-[(2-methacryloyl)ethyl(p-boronic acid benzyl) dimethylammonium bromide] (BA-PDMAEMA, BAP), to ensure release in the ROS-rich environment of AD-affected neurons. Intranasally administered, these engineered nanoparticles REXO/BAP/siRNAs navigated to the diseased brain regions, fusing with neuronal membranes for controlled release of siRNA in high-ROS environment, reducing BACE1 and caspase-3 levels, and thus Aβ plaques and neuronal apoptosis, as illustrated in Fig. 4a. Additionally, these MSC exosomes decreased reactive astrocyte numbers, collectively mitigating AD pathology and cognitive deficits.

Similarly, Iyaswamy et al. established a novel, exosome-based targeted drug delivery system for AD [243]. They engineered hippocampus neuron cell-derived exosomes with overexpression of Fe65, which can interact with APP-overexpressed neuron cells in the brains of AD mice. The engineered EV carries Corynoxine-B (Cory-B), an autophagy inducer, have been found to be effective in AD and PD. Cory-B can decrease the level of APP by inducing autophagy in neuronal cells. The Fe65-overexpressing HT22 hippocampus neuron cell-derived exosomes (Fe65-EXO), loaded with Cory-B (Fe65-EXO-Cory-B), capitalize on the disrupted Fe65-APP signaling to ensure APP-targeted delivery of Cory-B. Notably, treatment with Fe65-EXO-Cory-B successfully induced autophagy within APP-expressing neuronal cells, which was crucial in attenuating cognitive decline and the underlying pathogenesis associated with AD. A growing number of studies have explored the use of RNA-based therapeutics, specifically siRNA and miRNA, as therapeutic payloads for treating brain disorders. These nucleic acids are encapsulated within EVs to leverage their natural delivery capabilities.

EVs have also been investigated as theranostic targets for CNS disorders to achieve their therapeutic intervention and diagnostic assessment simultaneously. Perets et al. have developed a technique for the longitudinal and quantitative in vivo neuroimaging of exosomes, utilizing classic X-ray CT coupled with gold nanoparticles (GNP) as contrast agents, as seen in Fig. 4b [244]. The research team was able to noninvasively monitor the migration and homing behaviors of MSC-exo when administered intranasally to murine models. This innovative approach was applied across a spectrum of brain pathologies, including stroke, autism, PD, and AD. This method highlights the potential of MSC-exo as a versatile tool for targeting and treating a variety of neurological conditions, showcasing their ability to navigate to specific areas of the brain affected by disease. The study revealed that in models of brain pathology, MSC-exo exhibited a specific targeting and accumulation within the affected regions, persisting for up to 96 h post-administration. On the contrary, healthy control models showed a more diffuse migration pattern of MSC-exo, with clearance observed within 24 h. A strong correlation was identified between the neuro-inflammatory signal and MSC-exo accumulation, indicating that the exosomes’ homing ability is likely driven by inflammatory processes. An intriguing finding is that MSC-exo displayed a selective uptake by neuronal cells located in the pathological regions, while glial cells did not exhibit this uptake. These findings suggest a targeted mechanism of action for MSC-exo, with significant implications for their use in theranostic applications in various brain pathologies. Nanoparticle-assisted in vivo neuroimaging of exosomes has been widely studied by others as well [245, 246].

### PD

The diagnosis of PD is reliant on clinical motor symptoms and neuroimaging; however, by the onset of symptoms, it is estimated that 50–70% of substantia nigra pars compacta dopaminergic neurons have already been lost [247, 248]. The need to diagnose PD at an earlier stage, before this irreversible neuronal damage occurs, is imperative. Because EVs generated from CNS cells contain pathogenic misfolded proteins and dysregulated miRNAs that contribute to the progression of neurodegenerative disorders, they are considered noninvasive biomarkers for the early diagnosis of Parkinson’s disease.

Due to the relevance of α-syn in PD pathology as well as the proximity of CSF to the CNS, the quantification of α-syn in EVs isolated from patient CSF is of great interest for PD diagnostics. Stuendl et al. characterized α-syn in the sEVs of patients with α-syn related neurodegenerative diseases and found that α-syn levels were altered in patients with various stages of PD. Additional experiments were conducted, and these experiments showed that sEVs isolated from CSF can induce the oligomerization of soluble α-syn in an in vitro model [249]. This study established α-syn in CSF-derived sEVs as a relevant biomarker in PD and an important propagator of PD pathology. More recently, Herman et al. determined that α-syn levels in CSF-derived sEVs were upregulated in a small patient cohort with PD compared to control patients with non-synucleinopathies neurological disorders. Subsequently, CSF-derived sEVs isolated from PD patients were intranasally injected into healthy mice, resulting in the in vivo aggregation of α-syn in these mice and the onset of PD-like symptoms [250]. It is apparent that EVs present in the CSF of PD patients contain abnormal levels of relevant biomolecules, such as α-syn, and participate in the spread of neurodegeneration. However, due to the relatively invasive nature of CSF collection via lumbar puncture, the analysis of EVs present in other biofluids for PD diagnosis is of growing interest.

In a study evaluating α-syn levels in EVs isolated from blood plasma, Stuendl et al. determined that α-syn concentration was higher in PD patients (*n* = 94) compared to healthy controls (*n* = 50). Similarly, plasma-derived EV α-syn concentration was observed to be significantly higher in patients diagnosed with PD compared to patients diagnosed with dementia with Lewy bodies (DLB) and progressive supranuclear palsy (PSP), which is a neurodegenerative disease not related to α-syn and characterized by Parkinson’s syndrome. Regarding diagnostic capability, the α-syn concentration in plasma-derived EVs could distinguish between PD and healthy controls with an AUC of 0.769 [251]. Besides blood-derived EV protein concentrations for PD diagnosis, the expression of miRNAs in these exosomes may also serve to non-invasively diagnose PD.

A recent work by He et al. used RNA sequencing to identify differentially expressed miRNAs in the serum EVs of patients with varying stages of PD (*n* = 72) compared to healthy controls (*n* = 31). Of these differentially expressed miRNAs, six serum EV-derived miRNAs were further validated by receiver operating characteristic (ROC) curves and qRT-PCR in an additional cohort of 40 participants. The research concluded that hsa-miR-374a-5p, hsa-miR-374b-5p, hsa-miR-199a-3p, hsa-miR-28-5p, hsa-miR-22-5p, and hsa-miR-151a-5p could be potential biomarkers in serum EVs for PD prognosis and diagnosis [153]. More recent works involving serum EVs for PD diagnosis incorporate advanced technologies, such as nanomaterials, for improved diagnostic sensitivity and selectivity. In research by Fu et al., zwitterionic pCBMA polymer brush-coated magnetic beads (MBs) functionalized with anti-L1CAM were employed for the specific capture or neuron-derived EVs. Anti-α-syn and anti-Synt-1 antibodies were immobilized on a gold electrode, and electrochemical impedance spectroscopy was used to detect the two protein targets from EV lysates. This method determined up-regulated levels of the pathological protein α-syn in the EVs of PD patients (*n* = 20) versus healthy controls (*n* = 20). The new nanotheranostic platform displayed improved sensitivity, with a fivefold reduced necessary sample volume for analysis compared to an electrochemiluminescence kit [252]. More recently, the same group again captured neuron-derived EVs from blood serum using zwitterionic polymer-coated MBs functionalized with anti-L1CAM. The serum samples and MBs were agitated inside a microfluidic device using an alternating current magnetic field, enabling efficient EV capture. Subsequent EV lysis and detection of α-syn via a standard electrochemical sandwich ELISA distinguished PD patients (*n* = 20) and healthy controls (*n* = 20) with an AUC of 0.78 [253]. Incorporating nanomaterials allowed for rapid and specific isolation of an EV subpopulation in serum and the detection of a PD-related EV biomarker.

Besides their diagnostic potential, EVs are also gaining attention as drug-delivery vehicles for PD therapeutics. There are two main directions for EV therapeutics in PD treatment: (1) the direct use of native EVs released from stem cells and (2) the engineering of EVs to deliver biologics, small molecule drugs, etc. Stem cell-derived EVs have demonstrated similar neuroprotective, anti-inflammatory, and neurogenic properties as their parent cells, making them optimal particles for PD treatment [212]. A study by Narbute et al. employed the intranasal administration of EVs derived from human exfoliated deciduous teeth stem cells (SHEDs) into a 6-hydroxydopamine (6-OHDA) medial forebrain bundle (MFB) rat model of PD. Following intranasal administration of SHED-derived EVs, the 6-OHDA-treated rats demonstrated improved motor functions and increased tyrosine hydroxylase expression in the striatum and substantia nigra. Narbute et al. hypothesized that the presence of Cu/Zn SOD1 and other antioxidant proteins in the SHED-derived EVs could combat the detrimental effects of 6-OHDA-induced oxidative stress on dopaminergic neurons [254]. Other recent works focused on mesenchymal stem cell-derived sEVs for PD therapeutics. Xue et al. demonstrated the therapeutic potential of EVs isolated from MSCs for promoting angiogenesis of human brain microvascular endothelial cells (HBMECs) [255]. The improvement of the neurovascular unit may serve as a viable treatment for PD. Chen et al. used sEVs isolated from human umbilical cord MSCs (hucMSCs) to promote the proliferation and induce autophagy of 6-OHDA-stimulated SH-SY5Y cells in an in vitro PD model [256]. In the Parkinson’s disease rat model, isolated sEVs were injected into the tail vein. These sEVs, which were derived from hucMSCs, successfully pierced the BBB. As a result, the loss of dopaminergic neurons in the substantia nigra decreased significantly, whereas dopamine levels in the striatum increased. Moreover, the PD model rats exhibited improved behavioral function following sEVs injection, indicating the potential of this therapeutic strategy [256].

Despite the demonstrated potential of native EVs to treat PD, researchers have achieved enhanced therapeutic effects by combining these endogenous EVs with exogenous materials in advanced feats of EV engineering. A principal interest in EV engineering is the targeted delivery of active biologics, such as antisense oligonucleotides (ASO) and therapeutic miRNAs. These nucleic acids have great potential for silencing target genes involved in PD pathology and neuroprotective properties; however, their rapid degradation in vivo by endonucleases limits the clinical utility of these biologics [257]. Researchers recently loaded these nucleic acids inside sEVs to provide physical protection and targeted delivery of these therapeutics to the brain. Yang et al. loaded ASO targeting the human SNCA gene into human bone marrow mesenchymal stem cell-derived sEVs to attenuate α-syn aggregation in vitro and in vivo. Upon delivery of these EV-loaded ASOs to the brains of an α-syn A53T transgenic mouse model, α-syn mRNA, and protein levels decreased in the substantia nigra accompanied by the improvement of motor function [258]. Li et al. [259] and Esteves et al. [260] have loaded non-coding RNAs, such as miRNAs, demonstrating therapeutic potential for EVs by transfection of parent cells or by direct transfection of isolated EVs, respectively. Besides nucleic acids, researchers have also enriched EVs with biologics such as proteins for PD therapy. Zhao et al. used EVs derived from macrophages transfected with GDNF to treat Parkinson’s disease. GDNF is known to increase neuronal survival. GDNF-enriched EVs treated with transgenic Parkin Q311 (X) Intranasal injection of a mouse enhanced neuronal survival, decreased neuroinflammation, and improved motor performance in the Parkinson’s disease animal model [261].

**Fig. 5:**
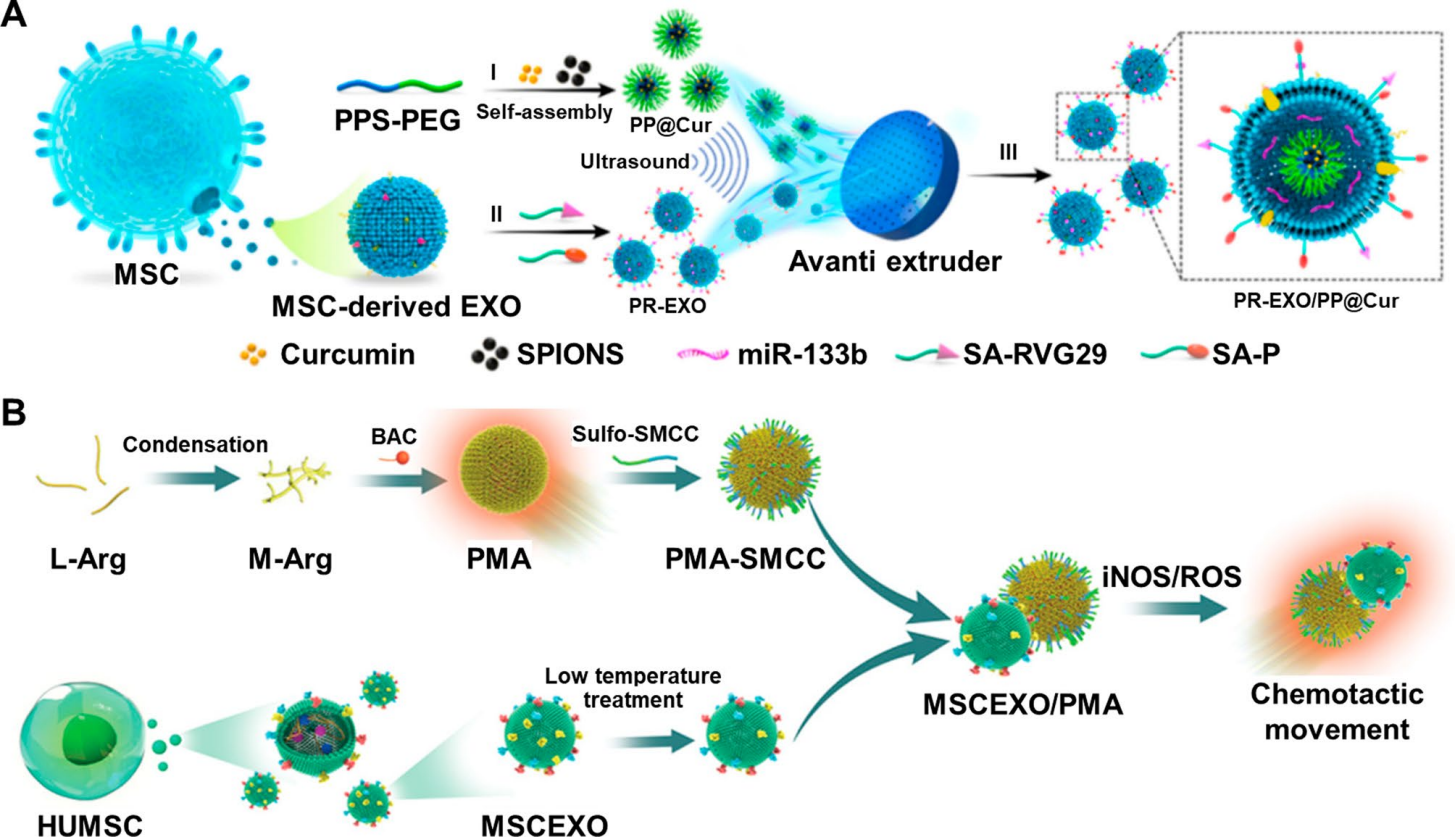
Engineered EV constructs for treatment of PD. **(A)** Schematic of PR-EXO/PP@Cur preparation. (I) (ROS)-responsive amphiphilic polymer PPS − PEG, curcumin and SPIONs were self-assembled into a micellar core. (II) MSC-derived sEVs were decorated with penetratin (P) and rabies virus glycoprotein (RVG29) peptides via hydrophobic carbon chains to form PR-EXO. (III) The inner micellar core and outer PR-EXO layer were combined by extrusion to construct the PR-EXO/PP@Cur. Reproduced with permission from [262]. Copyright © 2022, American Chemical Society. **(B)** Schematic of EV engineered with artificial module by conjugating L-arginine (L-Arg) with methacrylate anhydride (MA) to form arginine methacrylate (M-Arg). After crosslinking with BAC, zwitterionic nanoparticles poly (meth-acrylate arginine) (PMA) were prepared. Cross-linking agent sulfo-SMCC was reacted with PMA to form PMA-SMCC, which was subsequently combined with HUMSC derived EVs to form MSCEXO/PMA capable of migrating to the upregulated ROS and iNOS in the PD microenvironment. Reproduced with permission from [263]. Copyright © 2022 Wiley-VCH GmbH

In addition to engineering EVs as nanocarriers for the delivery of biologics to the brain, great efforts have been made to combine EVs with nanotechnology for multimodal PD therapeutic platforms [262, 263]. A notable example of this was published by Peng et al., in which a ROS-responsive amphiphilic polymer poly(propylene sulfide)-polyethylene glycol (PPS − PEG) micellar core was loaded with curcumin and superparamagnetic iron oxide nanoparticles (SPIONs)., MSC-derived sEVs were decorated with penetratin (P) and RVG 29 peptides and co-extruded with the Subsequently micellar core to construct the PR-EXO/PP@Cur nanotherapeutic as depicted in Fig. 5a. The modification of the extracellular vesicle (EV) shell with specific peptides facilitated its crossing of the BBB and directed the PR-EXO/PP@Cur complex towards the lesion site. The PPS-PEG component underwent degradation within the lesion site, characterized by a high ROS microenvironment. This degradation process triggered the controlled release of curcumin, a therapeutic agent, at the target site. Additionally, the incorporation of SPIONs within the complex served a dual purpose: they not only contributed to the therapeutic strategy but also acted as MRI contrast agents. This feature allowed for the non-invasive tracking of the EVs, providing real-time insights into their distribution and the dynamics of drug release within the brain. In a PD mouse model, the PR-EXO/PP@Cur decreased the concentration of α-syn aggregates and increased the amount of dopaminergic neurons in the substantia nigra, and improved the motor function of the PD mice [262]. Recently, Wang et al. combined native EVs with an independent artificial module enabling the chemotactic ability of their therapeutic to the PD microenvironment. After the synthesis of poly (methacrylate arginine) zwitterionic nanoparticles, these artificial modules were combined with HUMSC-derived EVs as illustrated in Fig. 5b. The resulting nanotherapeutic showed the ability to improve motor function and reduce neuroinflammation in vivo [263]. With the ongoing advancements in EV engineering techniques, the effective treatment of PD may one day be realized.

### Other (ALS and HD)

**Fig. 6:**
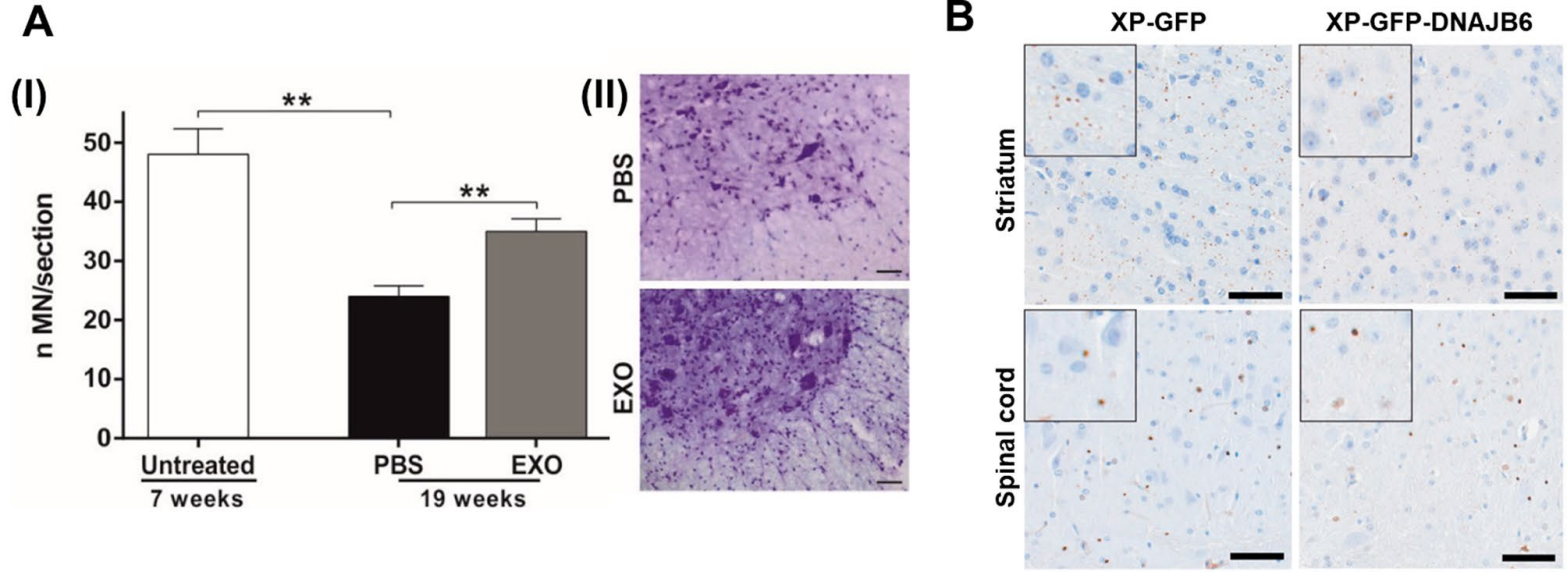
Native EVs and engineered EV constructs and therapeutic effects in ALS, HD, and ischemic stroke. **(A)** Improved motoneuron survival following administration of ASC-derived EVs quantified by (I) a stereological count of lumbar motoneuron and (II) Nissl staining. Reproduced with permission from [274]. Copyright © 2020 by the Authors. **(B)** Immunohistochemistry images of the striatum and spinal cord of R6/2 mice showing a decrease in HTT aggregates (brown spots) upon administration of XP-GFP-DNAJB6. Scale bar: 50 μm. Reproduced with permission from [280]. Copyright © 2021 by the Authors

Less common neurodegenerative diseases such as ALS still pose a major burden to afflicted patients and their families. Accurate diagnosis of ALS is complicated by the unreliable biomarkers and the heterogeneous nature of the disease [264]. In order to improve early diagnosis of ALS in a non-invasive manner, interest is growing in liquid biopsy biomarkers for disease detection [264–266]. Particularly, due to the nature of EVs and their superior cargoes and stability in biofluids, EVs have garnered great interest in the non-invasive diagnosis of ALS [267–272]. When it comes to EVs as biomarkers for ALS, many researchers have focused on dysregulated miRNAs [273]. Banack et al. confirmed previous studies reporting that miR-146a-5p is up-regulated while miR-4454 is down-regulated in the blood plasma-derived EVs of ALS patients compared to healthy controls [267]. EVs are also being investigated as therapeutic moieties for ALS treatment, and these EVs are typically isolated from stem cells [274, 275]. In a study by Bonafede and colleagues, sEVs were isolated from adipose-derived stem cells (ASCs) and treated to a (SOD1(G93A)) ALS mouse model via either intravenous injection or intranasal administration. ASC-derived EVs demonstrated beneficial therapeutic effects in vivo, such as improved motor function and decreased glial cell activation. Also, as seen in Fig. 6a, intravenous injection of ASC-derived EVs increased motoneuron survival, as indicated by Nissl staining [274]. It is likely that EV engineering may improve the prospects of ALS treatment through enhanced targeting and delivery of pharmacological drugs and/or biologics.

Due to the autosomal dominant nature of HD and the genetic cause, HD can be unequivocally diagnosed by genetic testing. However, researchers have still established promising biomarkers in CSF and blood for HD diagnosis, such as mHTT and NFL [276]. The identification of EV-associated biomarkers for HD is sparse. However, recent research by Anabeh et al. demonstrated that Huntingtin (HTT) co-isolates with plasma-derived sEVs in transgenic (TgHD) and knock-in (KI-HD) porcine models and human HD patient samples. Moreover, they reported the differential expression of HTT and mHTT in the EV samples, indicating the potential of EVs as disease biomarkers [277]. More commonly, researchers are investigating the therapeutic potential of EVs for HD. Researchers have employed EVs isolated from ASCs and transfected HEK 293 for the treatment of HD in vitro and in vivo [278, 279]. In recent years, researchers have employed sEVs to deliver therapeutics targeting mHTT mRNA or protein. In their investigation, Joshi and colleagues introduced the molecular chaperone DNAJB6b to sEVs generated from NSCs. They then conducted experiments both in vitro and in vivo to demonstrate the decrease in polyglutamine aggregation [280]. C17.2 cells were transfected with a GFP-DNAJB6-encoding plasmid, and the DNAJB6-enriched EVs decreased polyglutamine aggregation in EGFP-Htt(Q74) HEK293T cells. Moreover, the DNAJB6-enriched EVs reduced HTT aggregates by 31% in the brains of R6/2 HD transgenic mice, as illustrated by immunohistochemistry images in Fig. 6b showing fewer aggregates (brown spots) [280]. To suppress mHTT protein formation prior to translation, Zhang et al. employed a short-interfering RNA (siRNA) that was self-assembled in vivo for the reduction of mHTT [281]. In their study, a cytomegalovirus promoter-directed genetic circuit encoding a neuron-targeting RVG tag and a mHTT siRNA was injected into mice, uptaken by liver cells, and self-assembled into EVs. The endogenous EV-circulating system delivered the engineered EVs to the brain, reducing the levels of mHTT in the cortex and striatum. The monogenic nature of HD may allow engineered EVs to provide effective targeted therapy in the future.

### TBI

The diagnosis of severe TBI traditionally relies on clinical assessments and imaging studies. However, these methods have major difficulties in reliably detecting chronic TBI, owing to the lack of molecular biomarkers and intrinsic limitations in current imaging technologies. To address this gap, Ko et al. have innovated a microchip-based diagnostic tool to offer a more comprehensive characterization of TBI by analyzing RNA contained within brain-derived EVs [282]. This novel approach leverages a targeted panel of EV-associated miRNAs, which are analyzed using advanced machine learning algorithms to delineate the nuanced states of brain injury and recovery. In this study, brain-derived EVs are selectively isolated through a method utilizing surface marker-specific nanomagnetic particles, ensuring the high purity of the sample. Following isolation, the RNA content of these EVs undergoes sequencing, facilitating the discovery of potential biomarkers that reflect the molecular underpinnings of TBI. The subsequent application of machine learning algorithms to process the miRNA profiles from the EV cargo enables a nuanced, minimally invasive assessment of the TBI state.

NSCs have been explored as a potential therapeutic avenue for replacing neurons lost to CNS disorders [283]. Despite their promise, the clinical application of NSC therapy faces significant hurdles, including immune rejection in the absence of substantial immunosuppression, low rates of cell engraftment, teratoma formation risks, and the invasiveness of intracerebral transplantation. Recent investigations have illuminated the role of EVs produced by NSCs, astrocytes, and microglia in both the physiological responses and pathological alterations associated with TBI [189]. These EVs are recognized for their capacity to mediate neuroregulatory and reparative functions, offering a compelling foundation for developing innovative regenerative therapies, namely cell-free therapies [175]. Notably, the exosomes from these cells are rich in miRNAs and proteins that support neuroregeneration, inhibit apoptosis and inflammation, provide antioxidant benefits, and repair the BBB [114]. Furthermore, these exosomes harbor miRNAs and proteins critical for synaptic plasticity and cognitive function enhancement [202], marking them as a potent strategy for cell-free regenerative medicine in CNS disorders.

**Fig. 7:**
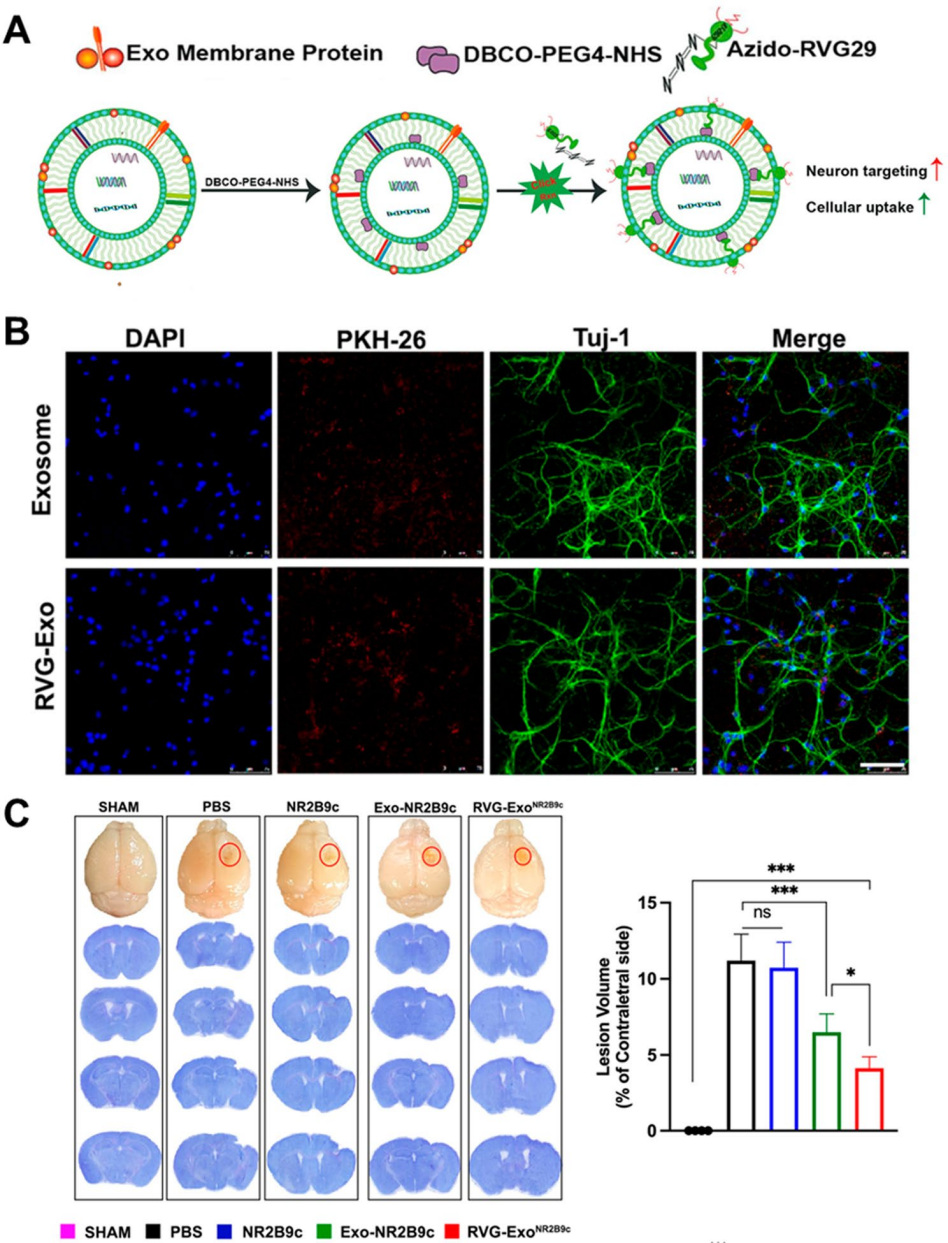
EV-based nanotheranostics for TBI. **(A)** A schematic representation of RVG29 decorating on the exosome surface using click chemistry. **(B)** Fluorescence images of primary neurons uptake RVG29 decorated exosomes. Scale bar = 75 μm, and **(C)** Representative images of whole brain and cresyl violet staining after controlled cortical impact insult for lesion volume check in different treatment groups (Left) and quantification of lesion volume. (Right). Reproduced with permission from [286]. Copyright © 2023 Elsevier B.V. All rights reserved

Recently, engineered EVs with nanotechnology have been investigated to maximize the nanotheranostic potentials. RVG 29 modification can significantly improve the targeting capability of the EVs toward neurons both in vivo and in vitro, compared to native EVs [284, 285]. Haroon et al. have made a significant advancement in TBI treatment by engineering exosomes derived from the microglia cell line BV2 [286], as illustrated in Fig. 7a. These exosomes were conjugated with RVG 29 to enhance their neuron targeting efficiency, a critical factor for effective TBI therapy [Fig. 7b]. the engineered exosomes were also loaded with NR2B9c, a compound known for its neuroprotective properties, offering a promising therapeutic effect for TBI. This combination of using naturally occurring nano-sized exosomes as a brain cargo delivery system, RVG29 as a neuron-targeting agent, and NR2B9c as a neuroprotective agent constitutes a novel and clinically viable strategy for TBI treatment [Fig. 7c].

For treating TBI, EVs have been administered intravenously or via direct injection. Recent advancements in sustained-release bio-nanomaterials have further optimized these delivery methods. Studies have integrated EVs with nano bioscaffolds, such as polypeptide hydrogels and ECM, to repair damaged tissues effectively [287–289]. These composites facilitate a controlled, steady release of exosomes at injury sites. Moreover, EVs, due to their nanoscale size, offer unique advantages in being easily combined with other biomaterials to achieve synergistic therapeutic effects. A notable application of this strategy involves the use of biological scaffolds combined with EVs derived from HUCMSCs, specifically engineered for in situ inductive recovery. In a pioneering study by Liu et al., exosomes from BDNF-stimulated HUCMSCs (BMExos) were incorporated into collagen/chitosan scaffolds via 3D printing [290]. These scaffolds, which have outstanding mechanical properties and biocompatibility, were shown in vivo to significantly improve neuromotor and cognitive abilities in a rat TBI model, demonstrating the promise of integrating exosomes with advanced bio-nanomaterials for neural repair.

### SCI

Numerous studies focusing on spinal cord injury (SCI) have consistently proven that the application of either native stem cell-derived extracellular vesicles (EVs) or engineered EVs in SCI mice plays a crucial role in facilitating a significant repair of the injury. This is comparable to the therapeutic effects of stem cell transplantation, but without the accompanying drawbacks associated with cell transplantation [291]. A growing number of studies suggest EVs derived from various cell lines, such as NSCs and MSC are neuroprotective, angiogenic, and immunomodulatory. Furthermore, they can easily cross the blood-spinal cord barrier and, therefore, can promote axonal regeneration [292–294]. Combined with the advantages of EVs, nanomaterials for molecular targeted drug delivery are expected to become the next generation of intelligent engineering biomaterials for precision medicine. Kim et al. engineered iron oxide nanoparticle (IONP)–incorporated exosome-mimetic nanovesicles (NV-IONP) from IONP-treated hMSCs and evaluated their therapeutic efficacy in a clinically relevant model for SCI [295].

**Fig. 8:**
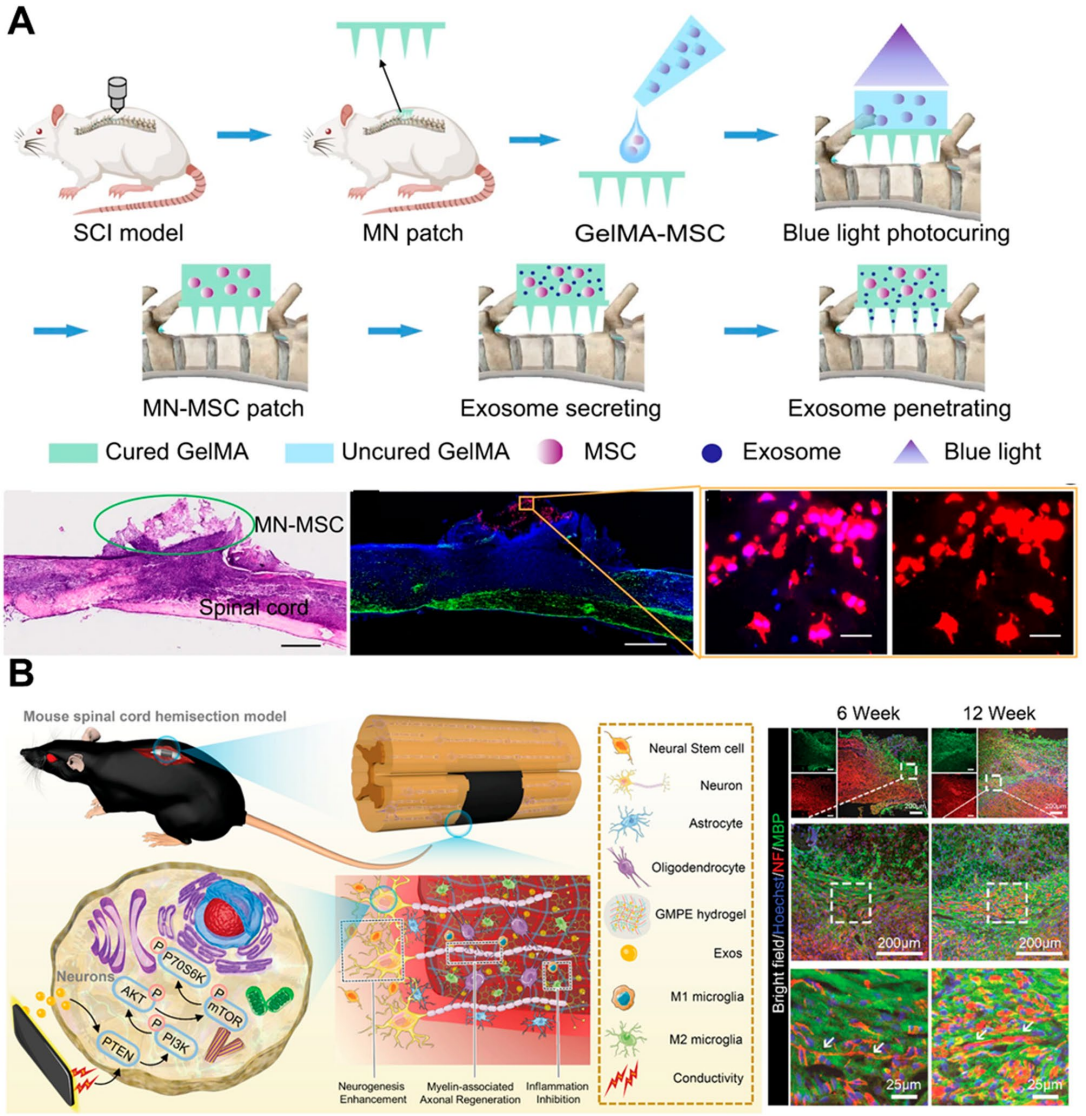
EV-based nanotheranostics for SCI. **(A)** Schematic illustration of MN-MSC patch implantation on the injury site of the spinal cord (Top), and HE staining of the injured spinal cord with MN-MSC patch implantation (Bottom left), representative images of immunohistochemical staining for MN-MSC patches on the injured spinal cord on day 7 after SCI (Bottom center), and high-magnification images of the boxed area (Bottom right). Reproduced with permission from [296]. Copyright © 2023, The Author(s). **(B)** The mechanism of GMPE hydrogel promoting SCI repair (Left) and myelinated axons regeneration evaluated at the 6- and 12-week time points (Right) Reproduced with permission from [299]. Copyright © 2022 The Authors. Advanced Science published by Wiley-VCH GmbH

IONPs have inherent magnetic properties that can be utilized for guided navigation, and these NV-IONPs were developed with the intention of improving the delivery of therapeutic molecules to the spinal cord. In a clinically relevant model of SCI, the systemic delivery of NV-IONP, directed by magnetic targeting, significantly increased their accumulation at the lesion site. This precise targeting led to several therapeutic outcomes. It promoted the formation of new blood vessels (angiogenesis), reduced inflammatory responses, and limited cell death by apoptosis. These combined effects resulted in a notable improvement in spinal cord functionality. This strategy highlights the potential of magnetic targeting to direct therapeutic agents to specific injury sites within the CNS, offering a promising approach for enhancing recovery in spinal cord injuries. The research conducted by Kim et al. highlights the potential of exosome-mimetic nanovesicles loaded with therapeutic agents and navigational tools to improve the treatment of spinal cord injuries. Fang et al. developed a novel porous microneedle (MN) array device designed to deliver EVs as a therapeutic intervention for SCI [296]. This innovative device combines MSCs with porous microneedles, offering a promising solution to the complexities of efficiently transporting EVs directly to injury sites on the spinal cord while minimizing tissue damage, as illustrated in Fig. 8a. To enhance the sustained delivery of MSC-derived EVs (MSC-EVs), the microneedle arrays are further equipped with a gelatin methacryloyl (GelMA) hydrogel, optimizing the device’s therapeutic potential for spinal cord repair.

EVs serve as nanosized carriers for drug and gene delivery in SCI treatments. Guo et al. have shown that MSC-Exo can be effectively administered intranasally, bypassing the BBB to reach the injured spinal cord site [297]. When these MSC-Exos are loaded with phosphatase and tensin homolog (PTEN) small interfering RNA (ExoPTEN), they specifically target and reduce PTEN expression in the SCI area. This targeted intervention not only suppresses PTEN expression but also significantly promotes axonal growth and neovascularization, while concurrently mitigating microgliosis and astrogliosis. In rat models of total spinal cord injury, the use of intranasal ExoPTEN treatment has shown a remarkable ability to restore structural and electrophysiological capabilities, and more importantly, it has been shown to facilitate substantial functional recovery.

Nanoscaffold biomaterial-based therapies, characterized by their adjustable modulus, topology, and patterned surfaces, have emerged as promising strategies for promoting neural tissue regeneration. These therapies offer three-dimensional matrices that provide the optimal biological, chemical, and physical properties to encourage cellular attachment, proliferation, differentiation, and neurite outgrowth. Hydrogels, owing to their soft, hydrated nature reminiscent of native nerve tissue, have gained popularity for fostering cellular growth and tissue formation post-SCI [298]. Fan et al. have innovated in this domain by developing exosome-loaded dual-network electroconductive hydrogels, integrating photo-cross-linkable gelatin methacrylate (GM) hydrogels with polypyrrole (PPy) hydrogels for the repair of SCI [299], as illustrated in Fig. 8b. These hydrogels encapsulate exosomes derived from bone marrow stem cells (BMSCs) through reversible interactions, preserving the exosomes’ structural integrity and bioactivity while enabling a controlled, sustained release upon implantation. This novel hydrogel system, GM/PPy/exosomes (GMPE), was evaluated using a mouse spinal cord hemisection model to assess its efficacy in supporting nerve regeneration and enhancing functional recovery post-SCI. The study represents a significant advancement in biomaterial-based neural regeneration strategies, potentially offering new avenues for SCI treatment through the synergistic combination of bioactive exosome delivery and conducive scaffold environments.

### Other (ischemic stroke)

Following ischemic stroke, diagnosis, evaluation of the extent of damage, and prompt therapeutic response are of paramount importance. While there are already existing methods for clinical diagnosis of ischemic stroke, it is important to note that traditional methods like non-contrast CT scans can sometimes complicate the quantification of infarcts. This is because the brain’s composition and density can vary, leading to potential confounding factors [300]. For this reason, researchers have explored other means for early and accurate stroke diagnosis. Circulating EVs have proven to be valuable biomarkers for the early, noninvasive diagnosis of ischemic stroke [301–304]. Zheng et al. reported that miR-124-3p, a negative regulator of inflammation, is decreased in the serum-derived EVs of acute ischemic stroke patients compared to healthy control patients [303]. More recently, Lu and colleagues found that the Silent Information Regulator 2 (SIRT2) protein is higher in the serum EVs of ischemic stroke patients compared to non-ischemic stroke patients [302]. These studies have demonstrated the potential of EVs in the diagnosis of stroke. Moreover, recent studies have established the utility of EVs in the treatment of ischemic stroke. Most studies examining the therapeutic potential of native EVs for ischemic stroke treatment involve mesenchymal stem cell (MSC)-derived and neural progenitor cell-derived EVs [305–307]. However, there is growing interest in EV engineering in treating ischemic stroke [308–310]. The delivery of therapeutic cargoes such as NGF mRNA [308] and circular RNA SCMH1 (circSCMH1) [309] via EVs has demonstrated improved neurogenesis and functional recovery, respectively, in animal models of stroke. EV engineering has also expanded beyond sEVs to include lEVs such as apoptotic bodies. Recently, You et al. utilized α-mangostin (α-M) to induce apoptosis of MSC while simultaneously loading the anti-inflammatory and neuroprotective α-M into the resulting apoptotic vesicles. For targeted delivery to the ischemic region of the brain, the apoptotic vesicles were surface-modified with matrix metalloproteinase (MMP) activatable cell-penetrating peptide. The engineered apoptotic vesicles demonstrated angiogenesis, cell proliferation, and improved functional recovery in vivo [310]. It is evident that EVs hold great promise for early diagnosis and treatment of ischemic stroke. Recent advances in nanotechnology-assisted EV diagnosis (Table 1) and therapy (Table 2) for CNS disorders are summarized in the tables.

**Table 1:** Nanotechnology assisted-diagnosis of neurological disorders using EVs

Disorders	Detection methods	EV sources	Biomarkers	Limit of detection	Accuracy	Fabrication methods	Ref
AD	SPR	Clinical human blood	Exosome-bound Aβ	200 exosomes	Distinguish from AD and MCI, HC and clincal groups	Double-layered gold plasmonic microarray with insoluble optical deposits for enzymatic amplification	[232]
AD	LSPR	Clinical human serum	Exosomal miRNAs: miRNA-125b, miRNA-15a, and miRNA-361	miRNA-125b: 10.54 aM miRNA-15a: 13.53 aM miRNA-361: 11.10 aM	99.52%	DNA-assembled advanced plasmonic AuNP architecture (DAPA)	[233]
AD	LSPR	Clinical human serum	Exosomal miRNAs: miRNA-125b, miRNA − 135a, miRNA − 15a, miRNA − 20a	miRNA-125b: 3.49 aM miRNA − 135a: 3.37 aM miRNA − 15a: 4.03 aM miRNA − 20a: 4.07 aM	98.22%	programmable curved plasmonic AuNP nanoarchitecture	[234]
AD	Field-effect transistor (EGFET)	Clinical human blood plasma	NFL from neuron derived exosomes	60 NDE/mL	Differentiate HC and patients at varying stages of AD	a nano-brush sensing structure, composed of vertically aligned high-aspect-ratio USNW coated with ITO	[238]
AD	Graphene electrolyte-gated transistor (G-EGT)	Clinical human serum	serum neuron-derived exosomal Aβ42(NDE-Aβ42)	447 ag/mL	100%	AuNP-decorated graphene electrolyte-gated FET	[237]
AD	Fluorescent biosensor	AD rat model	neurogenic exosomes-derived miRNA-193b and AβO42	miRNA-193b: 77 pM AβO42: 53 pM	n/a	A Graphene-based entropy-driven strand displacement reaction	[236]
AD	Electrochemical biosensor	Clinical plasma	Exosome bound-Aβ40 dimers	< 10 fg/mL	AUC = 0.85	a compact magnetic bead-based microelectrode array biosensor	[235]
PD	Electrochemical biosensor	Clinical human serum	Neuron-derived exosomal α-syn and Synt-1	n/a	n/a	Zwitterionic polymer coated molecular beads with anti-L1CAM functionality and antibody functionalized gold electrode	[252]
PD	Electrochemical biosensor	Clinical human serum	Neuron-derived exosomal α-syn and Synt-1	< pg/mL	AUC = 0.78	Zwitterionic polymer coated molecular beads with anti-L1CAM functionality and microfluidics	[253]
Stroke, autism, AD and PD	CT Brain imaging	Human bone marrow MSC	specifically targeted pathologically relevant murine models brains	n/a	n/a	GNP-labeled MSC exosome	[244]
TBI	Machine learning	TBI mice model and TBI patients	EV miRNAs: Upregulated: miR-129-5p, miR-212-5p, miR-9-5p Downregulated: miR-152-5p, miR-21, miR-374b-5p, miR- 664-3p	n/a	99%	Immunomagnetic isolation of brain-derived EVs using TENPO.	[282]

AD: Alzheimer’s disease, MCI: mild cognitive impairment, SPR: surface plasmon resonance, LSPR: localized surface plasmon resonance, AuNP: gold nanoparticle, NFL: neurofilament light chain, FET: field-effect transistors, AβO42: Aβ42 oligomers: AUC: area under the curve, MSC: mesenchymal stem cell, GNP: gold nanoparticle, TENPO: Track-Etched magnetic NanoPOre, USNW: upstanding silicon nanowires, ITO: indium tin oxide, CT: computed tomography, PD: Parkinson’s disease, L1CAM: L1 cell adhesion molecule

**Table 2:** Nanotechnology assisted-therapy of neurological disorders using EVs

Disorders	EV sources	Engineered components	Therapeutic outcomes	Ref
AD	dendritic cells from murine bone marrow	engineering the dendritic cells to express Lamp2b, GAPDH siRNA	Specifically deliver GAPDH siRNA to neurons, microglia, oligodendrocytes in the brain, resulting in a specific gene knockdown of BACE1.	[241]
AD	Mice MSC	BACE1 siRNA, Caspase-3 siRNA, Lesion-Recognizing NPs, neuron-specific RVG	Specifically deliver NPs to affected brain area, controllable release of siRNAs in high ROS environment, downregulate BACE1 and caspase-3 level, reduce the Aβ plaques and reactive astrocytes, inhibit neuron apoptosis	[242]
AD	HT22 mouse hippocampus neuron cel	Fe65-EXO loaded with Cory-B	Targeted delivery to APP-expressing neuron, Ameliorate cognitive decline and pathogenesis in AD mice	[243]
AD, PD, ischemic stroke and autism	hBM MSC	GNP-labeled MSC exosomes	specific targeting to pathological regions	[244]
PD	Human bone marrow MSCs	ASO	Reduce α-syn mRNA and protein levels in the SNc and improve motor function	[258]
PD	Mouse ADSC	miR-188-3p	Suppress autophagy and pyroptosis in vitro and in vivo	[259]
PD	hUCB-MNCs	miR-124-3p	Protect dopaminergic neurons in vitro and in vivo and improve motor function	[260]
PD	Mouse Bone Marrow Macrophages	GDNF	Increase neuronal survival, decrease neuroinflammation, and improve motor function	[261]
PD	MSCs	Curcumin, SPIONs, PPS − PEG, RVG29	Decrease α-syn aggregates and increase the amount of dopaminergic neurons in the SNc, and improve motor function in vivo	[262]
PD	HUMSCs	Zwitterionic NPs	Regulate microglia phenotype in vitro, improve motor function and reduce neuroinflammation in vivo	[263]
HD	Mouse NSCs	DNAJB6b	Decreased polyglutamine aggregation in EGFP-Htt(Q74) HEK293T cells, and reduce brain HTT aggregates in vivo	[280]
HD	Mouse liver cells	mHTT siRNA, RVG	Reduce the levels of mHTT in the cortex and striatum	[281]
TBI	microglia cell line BV2	neuroprotective peptide NR2B9c, RVG29	improve behavioral outcomes and reduce the lesion volume in a mice model	[286]
TBI	HUCMSCs	3D-printed collagen/chitosan scaffolds with exosomes	improve the regeneration of nerve fibres, synaptic connections and myelin sheaths in a TBI rat model	[290]
TBI	bone marrow MSC	hyaluronan-collagen hydrogel (DHC-BME)	Induce angiogenesis and neurogenesis, promote axonal regeneration, remyelination, synaptic formation and brain structural remodeling, improve spatial learning and memory, motor, sensory, reflex and balance in a TBI rat model	[288]
SCI	hBM MSC	IONP–incorporated exosome-mimetic nanovesicles	enhance blood vessel formation, attenuate inflammation and apoptosis, improve spinal cord function in a SCI mice model	[295]
SCI	hESC	A porous microneedle patch mounted with a gelatin methacryloyl (GelMA) hydrogel block	reduces the cavity and scar tissue formation, promotes angiogenesis, and improves survival of nearby tissues and axons in a SCI rat model	[296]
SCI	hBM MSC	PTEN siRNA GNP	enhance axonal growth and neovascularization, reduce microgliosis and astrogliosis, improve functional recovery in a SCI rat model	[297]
SCI	Mouse BMSC	exosome-loaded electroconductive hydrogels composed of GM and PPy	promote neuronal and axonal regeneration, and functional recovery at the early stage in an SCI mouse model.	[299]
Stroke	HEK293	NGF, RVG	Reduce inflammation and promote neurogenesis in vivo	[308]
Stroke	HEK293T	circSCMH1, RVG	Improve functional recovery, inhibit glial cell activation and immune cell infiltration, and upregulate transcription of MeCP2’s downstream genes in vivo	[309]
Stroke	HUMSCs	α-mangostin, MAP	Reduce inflammation and ROS in vitro. Increase angiogenesis, increase cell proliferation, and improve functional recovery in vivo	[310]

DC: dendritic cells, MSC: mesenchymal stem cell, RVG: rabies virus glycoprotein, Cory-B: Corynoxine-B, APP: amyloid-β precursor protein, GNP: gold nanoparticle, HUCMSCs: human umbilical cord mesenchymal stem cells, IONP: iron oxide nanoparticle, hESC: Human embryonic stem cell, BMSC: bone marrow stem cell, GM: gelatin methacrylate, PPy: polypyrrole, hBM: human bone marrow, ASO: antisense oligonucleotide, ADSC: adipose derived stem cell, hUCB-MNCs: human umbilical cord mononuclear cell, GDNF: glial-cell-line-derived neurotrophic factor, PPS − PEG: polymer poly(propylene sulfide)-polyethylene glycol, SNc: substantia nigra pars compacta, NSC: neural stem cell, EGFP: enhanced green fluorescent protein, HTT: huntingtin, mHTT: mutant huntingtin, siRNA: short-interfering RNA, ROS: reactive oxygen species, NGF: Nerve growth factor, circSCHM1: Circular RNA SCMH1, MeCP2: methyl-CpG binding protein 2, MAP: matrix metalloproteinase activatable cell-penetrating peptide, F56-EXO: Fe65-overexpression exosomes

## Conclusion and future perspectives

In summary, this review article describes an innovative approach for diagnosing and treating a broad range of CNS disorders. For diagnosis, this approach involves the analysis of EVs collected through lipid biopsy. We discuss a variety of CNS disorders, including AD, PD, TBI, SCI, ALS, HD, and ischemic stroke. Early and accurate detection of these disorders is crucial for improving patients’ quality of life. Thus, we examine the potential for using EVs for biosensing. EVs are tiny vesicles that transport proteins and miRNAs between cells. They play a vital role in cellular communication, whether beneficial or harmful. This feature makes EVs a promising candidate for diagnosing CNS disorders due to their potential to carry diagnostic markers. In addition, EVs produced from stem cells are well-known for their regenerative characteristics, which could make them a possible treatment for diseases of the CNS. In this review, we also highlight the significant role that EVs play in the pathophysiology of illnesses that affect the central nervous system. For example, in AD, EVs are key in transferring pathogenic proteins between neurons, showing their usefulness in disease detection by isolating protein-embedded EVs. Likewise, stem cell-derived EVs have regenerative potential, offering a promising treatment for conditions like TBI. Scientists have greatly improved their ability to harness the diagnostic and therapeutic potential of EVs due to the latest advances in technology and research methods. This rapidly growing field is currently witnessing an increasing number of clinical trials aimed at testing the clinical applications of EVs. These trials are crucial in advancing our knowledge of how EVs can be used to diagnose and treat neurological disorders. To provide a comprehensive overview of the current state of EV-based theranostic applications in neurology, we have compiled data on ongoing clinical trials in this area. Tables 3 and 4 provide a summary of EV-based diagnosis and therapy, respectively, with detailed information taken from ClinicalTrials.gov. This compilation not only reflects the breadth and diversity of research in this field but also highlights the potential of EVs as a novel diagnostic and therapeutic tool in neurology. Through these studies, researchers aim to unravel the complexities of EVs’ functions and their potential in clinical settings, paving the way for innovative treatments for various neurological conditions. However, despite the great potential of EVs as diagnostic markers and therapeutic vehicles, there are several challenges. These include issues with sensitivity, signal amplification for detection, selectivity, repeatability, stability, and therapeutic delivery efficiency. Addressing these challenges is essential to fully realize the potential of EVs in diagnosing and treating CNS disorders.

**Table 3:** Clinical trials of EV-based diagnosis for neurological disorders and injuries

Trial ID	Condition or Diseases	Study objective	Souce of EVs	Target enrollment	Status
NCT04603326	PD	Identify reliable markers of LRRK2 activity	human CSF	140	completed
NCT06082713	HD	discover blood-based biomarker for HD	Human blood	100	Recruiting
NCT05370105	Stoke	Evaluate EVs as prognostic biomarkers	Patient serume	100	Active, not recruiting
NCT05320250	PD	Analyze saliva EVs for PD diagnosis	Human saliva	180	Recruiting
NCT03775447	PD	Study LRRK2 activity in human CSF	Human CSF	38	completed
NCT01860118	PD	Investigate LRRK2 as PD marker	unknow	601	completed
NCT05645081	Stoke	Identify biomarkers associated with stroke	Endothelial	360	Recruiting
NCT05279599	TBI	Investigate EVs as TBI assessment marker	Human serum	300	unknow

**Table 4:** Clinical trials of EV-based therapies for neurological disorders and injuries

Trial ID	Condition or Diseases	Study objective	Souce of EVs	Administraion method	Target enrollment	Status
NCT03384433	Acute Ischemic Stroke	Promote neurovascular remodeling and functional recovery after stroke	MSC	Intravenous	5	Phase I Phase II
NCT04202770	Refractory depression, Anxiety disorders, Neurodegenerative diseases	Ultrasound delivery of exosomes for treatment of refractory depression, anxiety, and neurodegenerative dementias	Healthy, full-term Cesarean section amniotic fluid	Intravenous	300	Suspended
NCT06138210	Acute Ischemic Stroke	Evaluate safety and preliminary efficacy of exosomes derived from human iPSC in acute ischemic stroke	hiPSC	Intravenous	29	Phase I
NCT05886205	Refractory Focal Epilepsy	Evaluate the safety, tolerability, and preliminary efficacy of iPSC derived exosomes in the treatment of focal refractory epilepsy	iPSC	nasal drops	34	Early phase I
NCT05326724	Post-stroke Dementia	Explore the role of acupuncture-induced exosome in the treatment of post-stroke dementia	No information	Acupuncture	30	Not applicable
NCT04388982	AD	Evaluate the safety and efficacy of exosomes aerived from allogenic ASCs in subjects with AD	Allogenic ASCs	Not specified	9	Phase I Phase II

To overcome these obstacles, we suggest that nanotechnology can be a suitable alternative. Specifically, this is because nanotechnology offers a variety of tools that have the potential to successfully improve the characteristics of EVs, which in turn can significantly enhance their diagnostic and therapeutic capabilities. This review evaluates the integration of nanotechnology with EVs to develop CNS theranostic applications. A wide range of EV nanoengineering has been employed in this field, including nanomaterials and nanoplatforms. Nanomaterials have been integrated with EVs to enhance their therapeutic and diagnostic properties. For instance, noble metal nanoparticles have been used to improve the delivery of therapeutic agents to specific targets in the brain. The utilization of magnetic nanoparticles has proven to be effective in both isolating and imaging EVs. To improve the sensing performance of EVs, researchers have utilized graphene. Using hydrogels has proven to be highly effective in creating 3D scaffolds that imitate the characteristics of the ECM, thus playing a crucial role in promoting tissue regeneration. Nanopatterns and microfluidics have been used to isolate and purify EVs from complex biological samples. The collaboration between nanotechnology and EVs holds immense promise for advancing theranostic applications in CNS disorders. This approach has the potential to overcome many of the limitations of traditional drug delivery systems and diagnostic tools, such as poor bioavailability, toxicity, and low sensitivity. Overall, integrating nanotechnology with EVs represents a novel and exciting approach for the development of next-generation theranostics.

**Fig. 9:**
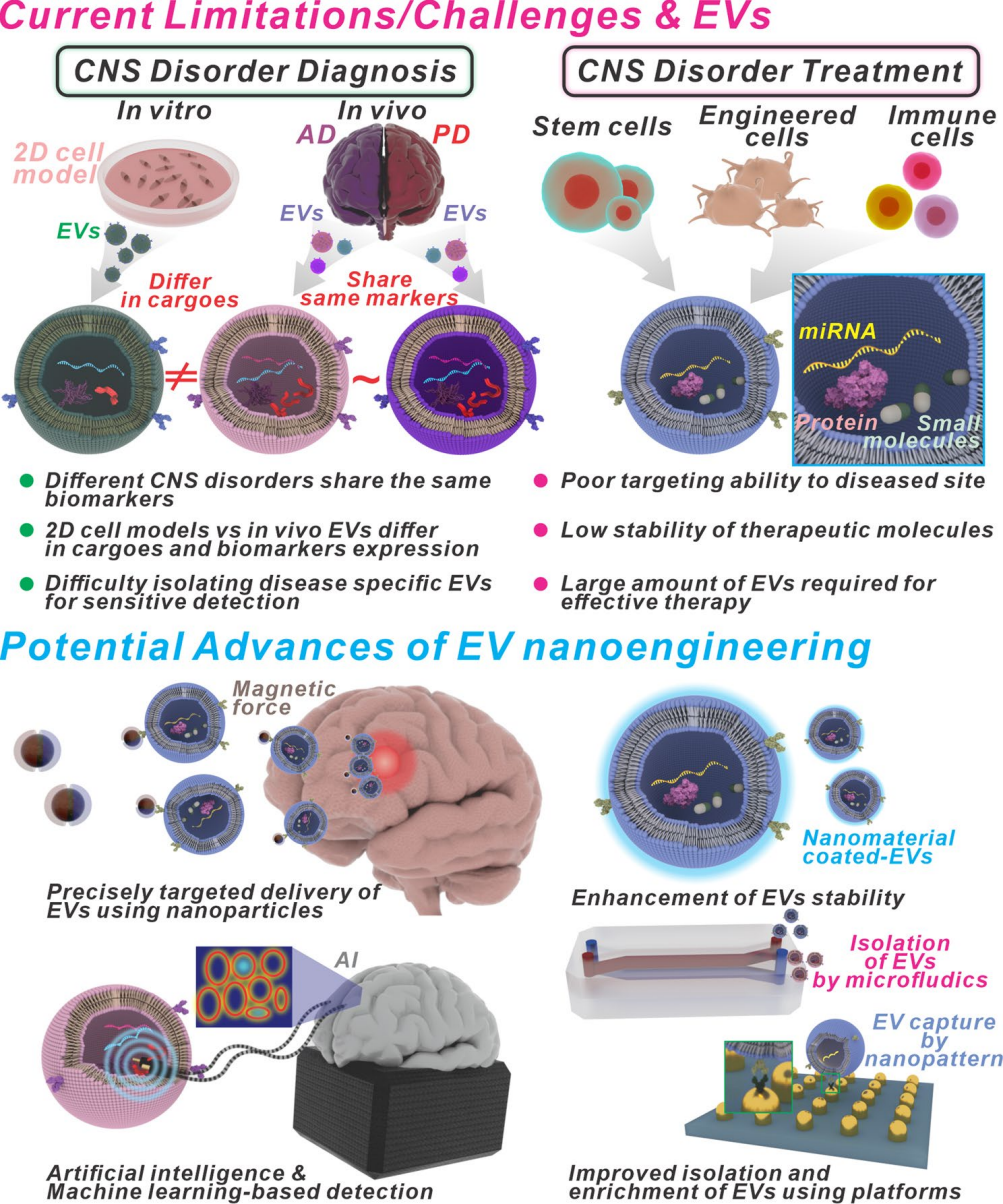
Current challenges and future perspectives of EVs for diagnosis and treatment of CNS disorders. The challenge of utilizing EVs for CNS disorder diagnosis is the overlapping biomarkers shared amongst differing CNS disorders such as AD and PD. Another challenge is the heterogeneity of EVs derived from in vitro cell culture conditions and those derived from in vivo patient conditions. The EVs derived from different origins contain different cargoes and biomarker expressions, thus complicating biomarker discovery and diagnosis. Moreover, the challenge of isolating disease-specific EVs complicates sensitive detection for diagnosis. The current challenges of utilizing EVs for the treatment of CNS disorders include the poor targeting ability of EVs to the diseased site, the low stability of therapeutic molecules associated with EVs, and the requirement of a large number of EVs for effective therapy. To combat these limitations, EV nanoengineering presents new avenues for improving the use of EVs in diagnosis and therapy. The combination of nanomaterials such as magnetic nanoparticles can enhance the precise targeted delivery of therapeutic EVs to the diseased site. Additionally, nanomaterial-coated EVs can enhance the stability of therapeutic molecules in EVs. To enhance diagnostic potential, the incorporation of Artificial intelligence (AI) and machine learning (ML) may improve accurate and large-scale diagnosis using EVs. Furthermore, microfluidics and nanopattern assisted EV capture can isolate disease specific EVs for sensitive diagnosis as well as enrich therapeutic EVs for effective therapy

While great progress has been made in EVs for CNS illnesses, unknown areas remain to be researched. [Fig. 9]. For instance, because of the incomplete understanding of the origins of CNS disorders, there is currently no foolproof method for the early detection of such disorders [311, 312]. Also, since many disorders share the same proteins or micro RNAs as markers, it is impossible to have selective detection of diseases [313–317]. To achieve the ideal detection of CNS disorders, particularly AD and PD, there needs to be further deep scientific investigation. It is necessary to understand the exact pathways for each disorder, and multiplex detection is required, not only detecting a single marker for diagnosis [318, 319]. Similarly, machine learning and artificial intelligence have been applied to this field to increase the precision of detection [320, 321]. Since computational methods can make work fast and efficient, it would be great for the early detection of CNS disease for large numbers of people. Another limitation is that in vitro and in vivo systems differ in terms of gene expression or protein profile. Even in the in vitro EVs, the EVs from 2D culture and 3D culture showed different miRNA amounts and species. To ensure the perfect diagnosis of CNS disorders by using EVs, we need to develop better in vitro cell culture models, such as co-cultured 3D cell-based platforms with vascular networks or brain assembloids [322–326]. In terms of the therapeutic utility of EVs, it is important to make EVs stable for a long time. To treat the EVs to a patient in the early stage for therapeutic purposes, the EVs should be ready in advance for immediate treatment. However, we cannot store the EVs for a long time since they lose their structure and bioactivity due to the destruction of the lipid layers [327–329]. Furthermore, a substantial quantity of EVs is needed to address CNS diseases, particularly in cases where the affected region is sizable. Although nanotechnology has the potential to improve the isolation efficiency of EVs, there is still a need for quantitative improvements in EV production [330–332]. To treat CNS disorders using EVs, EVs should only target the disease or disordered area cells, but even with nanotechnology, efficiency is quite low [333–336]. To overcome this, more precise targeting-engineered technology should be developed.

To conclude, this review presents a comprehensive and multidisciplinary examination of the utilization of EVs in the theranostics of CNS disorders. By marrying the principles of nanotechnology with the unique properties of EVs, promising methodologies have been unveiled to surmount the prevailing obstacles in both diagnosis and therapeutic intervention. Through collaborative and multidisciplinary research endeavors spanning diverse scientific domains, we can harness the full potential of EV-based theranostics. Such an integrated approach promises to enhance patient outcomes significantly and forge more productive strategies for addressing the myriad challenges posed by CNS disorders.

## Data Availability

The datasets used and/or analyzed during the current study are available from the corresponding author on reasonable request.

## Abbreviations

CNS: Central nervous system
AD: Alzheimer’s Disease
PD: Parkinson’s Dissease
TBI: Traumatic brain injury
ALS: Amyotrophic Lateral Sclerosis
MRI: Magnetic Resonance Imaging
PET: Positron Emission Tomography
Evs: Extracellular vesicles
mRNAs: messenger RNAs
HD: huntington’s disease
Aβ: amyloid-β
NFTs: intracellular neurofibrillary tangles
APP: transmembrane amyloid precursor protein
PSEN1: presenilin 1
PSEN2: presenilin 2
APOE: apolipoprotein E
CSF: cerebrospinal fluid
TDP43: TAR DNA-binding protein 43
UHDRS: Unified HD Rating Scale
TMS: total motor score
BBB: Blood-brain barrier
ROS: reactive oxygen species
CT: computed tomography
TNF: tumor necrosis factor
IL: interleukin
ECM: extracellular matrix
NSCs: neural stem cells
MSCs: mesenchymal stem cells
VEGF: Vascular endothelial growth factor
NGF: nerve growth factor
GDNF: glia-derived neurotrophic factor
BDNF: brain-derived neurotrophic factor
MVBs: multivesicular bodies
sEVs: small extracellular vesicles
lEVs: Large extracellular vesicles
qRT-PCR: quantitative reverse transcription polymerase chain reaction
miRNA: microRNA
p-Tau: phosphorylated Tau
ESCs: embryonic stem cells
DCs: dendritic cells
NK: natural killer
ESDR: entropy-driven strand displacement reaction
LSPR: localized surface plasmon resonance
DAPA: DNA-assembled advanced plasmonic architecture
SPR: Surface Plasmon Resonance
LNA: locked nucleic acid
MCI: mild cognitive impairment
GO: Graphene oxide
G-EGT: graphene electrolyte-gated Field-Effect Transistor
NFL: neurofilament light chain
NDEs: neuron-derived exosomes
USNW: upstanding silicon nanowires
EGFET: extended gate in a field-effect transistor
siRNA: short-interfering RNA
siRNA: small interference RNAs
MSC-exo: MSC-derived exosomes
GNP: gold nanoparticles
α-syn: α-synuclein
ROC: receiver operating characteristic
MBs: magnetic beads
SHEDs: exfoliated deciduous teeth stem cells
6-OHDA: 6-hydroxydopamine
MFB: medial forebrain bundle
SOD-1: superoxide dismutase 1
HBMECs: human brain microvascular endothelial cells
hucMSCs: human umbilical cord MSCs
ASO: antisense oligonucleotides
RVG: rabies virus glycoprotein
ASCs: adipose-derived stem cells
HTT: huntingtin
NV-IONP: nanovesicles containing iron oxide nanoparticles
GelMA: gelatin methacryloyl
GM: gelatin methacrylate
PPy: polypyrrole
BMSCs: bone marrow stem cells
GMPE: GM/PPy/exosomes
SIRT2: Silent Information Regulator 2
circSCMH1: circular RNA SCMH1
α-M: α-mangostin
MMP: matrix metalloproteinase
APEX: Amplified Plasmonic Exosome
ELISA: enzyme-linked immunosorbent assay
AFM: atomic force microscopy
L-Arg: L-arginine
M-Arg: arginine methacrylate
PMA: poly (meth-acrylate arginine)
MA: methacrylate anhydride
BAC: N,N’-bis (acryloyl) cysteamine
